# Modeling Normal and Pathological Ear Cartilage *in vitro* Using Somatic Stem Cells in Three-Dimensional Culture

**DOI:** 10.3389/fcell.2020.00666

**Published:** 2020-07-28

**Authors:** Eleonora Zucchelli, Martin Birchall, Neil W. Bulstrode, Patrizia Ferretti

**Affiliations:** ^1^Stem Cells and Regenerative Medicine Section, UCL Great Ormond Street Institute of Child Health, University College London, London, United Kingdom; ^2^UCL Ear Institute, University College London, London, United Kingdom; ^3^Department of Plastic Surgery, Great Ormond Street Hospital for Children NHS Foundation Trust, London, United Kingdom

**Keywords:** cartilage, development, ear, human, mesenchymal stem cells, microtia, spheroid cultures, tissue engineering

## Abstract

Microtia (underdeveloped ear) is a rare congenital dysmorphology affecting the development of the outer ear. Although human microtic cartilage has not been fully characterized, chondrogenic cells derived from this tissue have been proposed as a suitable source for autologous auricular reconstruction. The aim of this study was to further characterize native microtic cartilage and investigate the properties of cartilage stem/progenitor cells (CSPCs) derived from it. Two-dimensional (2D) systems are most commonly used to assess the chondrogenic potential of somatic stem cells *in vitro*, but limit cell interactions and differentiation. Hence here we investigated the behavior of microtic CSPCs in three-dimensional spheroid cultures. Remarkable similarities between human microtic cartilages from five patients, as compared to normal cartilage, were observed notwithstanding possibly different etiologies of the disease. Native microtic cartilage displayed poorly defined perichondrium and hyper-cellularity, an immature phenotype that resembled that of the normal developing human auricular cartilage we studied in parallel. Crucially, our analysis of microtic ears revealed for the first time that, unlike normal cartilage, microtic cartilages are vascularized. Importantly, CSPCs isolated from human microtic and normal ear cartilages were found to recapitulate many characteristics of pathological and healthy tissues, respectively, when allowed to differentiate as spheroids, but not in monolayer cultures. Noteworthily, starting from initially homogeneous cell pellets, CSPC spheroids spontaneously underwent a maturation process in culture, and formed two regions (inner and outer region) separated by a boundary, with distinct cell types that differed in chondrogenic commitment as indicated by expression of chondrogenic markers. Compared to normal ear-derived spheroids, microtic spheroids were asymmetric, hyper-cellularized and the inner and outer regions did not develop properly. Hence, their organization resembled that of native microtic cartilage. Together, our results identify novel features of microtic ears and highlight the importance of 3D self-organizing *in vitro* systems for better understanding somatic stem cell behavior and disease modeling. Our observations of ear-derived chondrogenic stem cell behavior have implications for choice of cells for tissue engineered reconstructive purposes and for modeling the etiopathogenesis of microtia.

## Introduction

Microtia (small ear) and anotia (absent external ear) are rare congenital defects affecting development of the outer ear (Luquetti et al., 2012; Cox et al., 2014). They can occur as part of a syndrome, such as hemifacial microsomia, Goldenhar syndrome, Treacher Collins, Nager, and CHARGE, or independently, with the mechanisms underlying the defect largely unknown (Luquetti et al., 2012). Different degrees of reduction in size and malformed shape are observed among microtic ears depending on the severity of the malformation, with anotia and the most severe cases of microtia requiring surgical reconstruction. Development of tissue engineering approaches would circumvent the use of autologous rib cartilage, currently the most successful reconstructive approach, but associated with high morbidity and in some cases respiratory complications (Ohara et al., 1997; Walton and Beahm, 2002; Kusuhara et al., 2009; Baluch et al., 2014; Pappa et al., 2014; Gu et al., 2018).

Notwithstanding much clinical interest in microtia, both normal and microtic ear cartilages remain under-characterized, and so are the stem/progenitor cells present in these tissues, which may be of value for autologous cell therapy (Anthwal and Thompson, 2016). Hence there is a need for carrying out a more systematic analysis of microtic cartilages both *in vivo* and *in vitro*.

It is well established that, although cartilage lacks intrinsic reparative ability, a unique population of cartilage stem/progenitor cells (CSPCs) is present in the superficial zone (SZ) of mature avascular cartilage and in the tissues immediately surrounding it (inner perichondrium) in both animals and humans (Engkvist and Wilander, 1979; Dowthwaite et al., 2004; Shirasawa et al., 2006; Togo et al., 2006; Kobayashi et al., 2011a, b; Kagimoto et al., 2016; Xue et al., 2016). These cells are thought to be involved in the maintenance of tissue homeostasis. CSPCs isolated from normal ear cartilage exhibit typical features of mesenchymal stem cells (MSCs), such as adherence to plastic, spindle-like morphology and three-mesenchymal lineage differentiation potential *in vitro* (Kobayashi et al., 2011a; Jiang and Tuan, 2015; Zhang et al., 2017).

Previous studies have demonstrated that CSPCs can be isolated also from the human microtic ear, and have the ability to proliferate and undergo chondrogenic differentiation; in addition, it has been proposed that microtic CSPCs can be used for cartilage reconstruction (Yanaga et al., 2009; Kobayashi et al., 2011a; Yanaga et al., 2012; Zhou et al., 2018). However, there are discrepancies on how “normally” microtic CSPCs behave, and studies directly comparing microtic cells with normal CSPCs from normal auricular cartilage or other sources are very limited. In addition, potential differences between normal and microtic cartilages *in vivo* have not been fully explored. A better understanding of microtic cells is important to fully establish their potential for cartilage engineering and may help to elucidate causes of the disease. It is also important to note that most studies of microtic cells have been carried out in 2-dimensional (2D) culture systems, that prevent the more complex cell interactions occurring in tissues (Laschke and Menger, 2017). Hence, we hypothesized that potential differences between normal and microtic ear cartilage may be obscured in standard 2D cultures but become apparent in 3D cultures where the cells are allowed to self-organize (spheroids).

To test this hypothesis, we assessed chondrogenic differentiation of microtic ear derived cells, both in 2D and in spheroid cultures, and compared them with chondrogenic cells derived from normal ear cartilage, and with other MSCs with chondrogenic differentiation ability, such as pediatric adipose-tissue derived stem cells (ADSCs). In parallel, we compared changes in human auricular cartilage with development and in microtic ears to gain further understanding of normal and microtic cartilage traits, and assess whether the spheroids modeled some aspects of the disease.

In addition to differences in cellularity and cytoarchitecture between healthy and microtic native cartilages, our analysis has demonstrated for the first time the presence of blood vessels in the chondrium layer of microtic cartilages. This is in contrast to healthy cartilages, that are always avascular, and identifies a new important landmark of the disease.

This study has also shown that following chondrogenic differentiation in 3D cultures, CSPCs derived from microtic ear cartilage remnants display differences in their spontaneous spatial organization as compared to normal ear CSPCs, which are not readily apparent in 2D cultures. Significantly, comparative analysis of differentiated spheroids and native cartilage has indicated that normal ear CSPC-derived spheroids display a structural organization resembling that of developing normal ear cartilage, including a chondrium layer and an inner and outer perichondrium (OP). In contrast, microtic ear CSPC-derived spheroids appear to reproduce some morphological features of pathological tissues, such as hyper-cellularization of cartilage nodules and disruption of the typical multi-layered architecture of cartilage suggesting they provide a suitable system for modeling the disease.

## Materials and Methods

All chemicals were from Sigma-Aldrich (United Kingdom), unless otherwise stated.

All procedures involving human tissue were carried out in accordance to the UK Human Tissue Act 2006.

### Human Fetal Ear Tissues

External ear tissues from human fetuses at different developmental stages used for tissue analysis were provided by a tissue bank, the Human Developmental Biology Resource^1^ (HDBR) under ethical approval (NRES Committee London – Fulham, United Kingdom). Dissected tissues were fixed in 4% PFA, dehydrated in ascending ethanol solutions, and embedded in paraffin using a Sakura Tissue-Tek TEC embedding machine (Sakura Tissue Tek). Sections (3 μm) were dewaxed in Histo-clear II (National Diagnostics, Atlanta, GA, United States) and then rehydrated by descending ethanol solutions, prior to histological staining and protein expression analysis by immunohistochemistry. Embryos at 16 and 22 post conception weeks (PCW) were used in this study.

### Human Pediatric Adipose and Ear Tissues

All abdominal adipose tissue and auricular cartilage used for tissue analysis and cell line generation (Supplementary Table 1), were collected from consenting patients under ethical approval from the Camden and Islington Community Local Research Ethics Committee (London, United Kingdom). Microtic ear tissues were obtained from surplus cartilage of patients undergoing autologous costal to ear graft reconstruction, whereas normal ear cartilage was obtained from a healthy donor, undergoing otoplasty as an aesthetic procedure. Dissected tissues were fixed in 4% PFA prior to cryo- or paraffin embedding and sectioning for histological staining and protein expression analysis by immunohistochemistry.

### Cell Growth and Differentiation

All cells were grown at 37°C with 5% CO_2_ in a humidified incubator.

#### Human Pediatric Adipose Tissue-Derived Stem Cells (ADSCs)

Adipose tissue-derived stem cells were isolated from lipoaspirates of pediatric patients as previously described (Guasti et al., 2012). Isolated ADSCs were cultured in high glucose Dulbecco’s modified Eagle’s medium (DMEM; Life Technologies) containing GlutaMAX^TM^ and supplemented with 10% embryonic stem cell-qualified fetal bovine serum (ES-FBS; ES-009-B, Merk Millipore, Burlington, MA, United States) and 1% penicillin/streptomycin.

#### Human Pediatric Normal and Microtic Ear Cartilage-Derived Cells (Normal and Microtic CSPCs)

Human normal and microtic CSPCs were derived using the primary explant technique, as previously described (Guasti et al., 2012). Explants and isolated cartilage precursor cells were cultured in DMEM containing GlutaMAX^TM^ and supplemented with 10% ES-FBS and 1% penicillin/streptomycin. After collection, all cartilages were extensively washed in phosphate buffer saline (PBS, pH 7.4) and cut into approximately 2 mm^3^ pieces following careful removal of fat and connective tissues. Two to three explants were plated onto each well of a 12 well culture plastic plate. Explants were allowed to adhere to the plastic for a few minutes before covering each of them with one drop of medium (approximately 100 μl). After 24–48 h, if the explants had attached to the plastic, more medium was added. Explants were handled carefully and fed very gently to prevent them from detaching and floating until the cells started to migrate out. Once the cells had become confluent, the explants were removed, and the cells were passaged and expanded in the same medium.

#### Adipogenic Differentiation

Adipogenic differentiation was induced in confluent cells by the addition of DMEM containing GlutaMAX^TM^, 1% penicillin/streptomycin, 10% ES-FBS, 10 ng/ml insulin, 500 μM 3-isobutyl-1-methylxanthine, 1 μM dexamethasone, and 1 μM rosiglitazone (Molekula, Gillingham, United Kingdom). After 3 weeks, cells were fixed in 4% PFA, and stained with Oil Red O working solution to visualize lipid droplets, as previously described (Guasti et al., 2012).

#### Chondrogenic Differentiation

Chondrogenic differentiation was induced in confluent cells by the addition of DMEM containing GlutaMAX^TM^, 1% penicillin/streptomycin, 10% ES-FBS, 0.1 μM dexamethasone, 50 μg/ml ascorbate, 10 ng/ml transforming growth factor β 1 (TGF β 1), and insulin-transferrin-selenium supplement (ITS, Sigma). After 3 weeks, cells were fixed in 4% PFA and stained with Alcian Blue dye for semi-quantitative analysis, as previously described (Guasti et al., 2012). Fold changes relative to chondrogenic measurements were calculated taking undifferentiated controls as reference (value 1).

#### Osteogenic Differentiation

Osteogenic differentiation was induced in confluent cells by the addition of DMEM containing GlutaMAX^TM^, 1% penicillin/streptomycin, 10% ES-FBS, 0.1 μM dexamethasone, 100 μg/ml ascorbate, and 10 mM β-glycerophosphate. After 3 weeks, cells were fixed in ice-cold 70% ethanol and stained with Alizarin Red solution as previously described (Guasti et al., 2012).

#### Spheroid Cultures

For 3D spheroid cell cultures, 3 × 10^5^ cells were resuspended in 100–150 μl of DMEM containing GlutaMAX^TM^, 1% penicillin/streptomycin, 10% ES-FBS, collected in 1.5 ml conical tubes with screw caps, allowed to settle down to the bottom of the tube for around 10 min, and centrifuged at 300 *g* for 5 min. Aggregates were incubated at 37°C in 5% CO2 overnight. After spheroid formation (usually within 24–48 h after centrifugation), the medium was replaced with either control or chondrogenic medium, and cells were fed 3–4 times a week. In each experiment at least four samples per treatment were used. After either 4 or 6 weeks, spheroids were fixed in 4% PFA prior to cryo-embedding and sectioning (10 μm sections) for histological staining and protein expression analysis by immunofluorescence.

#### Spheroid Diameter Measurement

Spheroids were imaged using a Leica MZ FL III Fluorescence Stereomicroscope (Leica) and their diameter measured using Fiji software. In undifferentiated spheroids that were less compact and had a less well-defined shape than the differentiated ones, the width was measured only considering the core of the samples, as shown in Figure 5A.

### Reverse Transcription-Polymerase Chain Reaction

RNA was extracted from cells and tissues using TRIzol reagent (Life Technologies) according to the manufacturer’s protocol. After treating with DNase I, Amplification Grade (Life Technology), RNA was retrotranscribed with Moloney murine leukemia virus reverse transcriptase (Promega, Madison, WI, United States). cDNA was amplified using GoTaq^®^ DNA Polymerase kit (Promega), in a Veriti^®^ Thermal Cycler (Applied Biosystems, Life Technology). Primers and conditions used are listed in Supplementary Table 2. No template controls containing water instead of cDNA were included in each PCR run. Glyceraldehyde 3-phosphate dehydrogenase (*GAPDH*) and 60s ribosomal protein L19 (*RPL19*) housekeeping genes were used as internal controls. After PCR amplification, the PCR product was analyzed by electrophoresis in agarose gels, with SYBR^®^ Safe DNA Gel Stain (10,000×) (Thermo Fisher Scientific) and visualized using a Gel Doc system (ChemiDoc^TM^ XRS + System, Bio-Rad).

### Western Blot Analysis

Proteins were extracted either from fresh or frozen cell pellets by homogenization for 15 min on ice in RIPA buffer (50 mM Tris-HCl pH 7.4, 150 mM NaCl, 1 mM phenylmethylsulfonyl fluoride (PMSF), 1 mM EDTA, 1% Triton X-100, 0.1% SDS, 1% sodium deoxycholate, containing one tablet of total protease inhibitor (cOmplete^TM^, Mini Protease Inhibitor Cocktail, Roche, Switzerland)/10 ml of buffer). The lysates were centrifuged at 11,000 *g* at 4°C for 15 min, and the supernatants containing the solubilized proteins were transferred to a new tube.

Whole protein extracts from porcine auricular cartilage were used as a positive control. Ears of adult pigs were collected from the Royal Veterinary College, University of London (Royal College Street, London, United Kingdom). First, pig auricles were extensively washed in 1× PBS and dissected to remove all skin and fat. The ear cartilage was then cut into stripes and snap–frozen in liquid nitrogen. Frozen cartilage was pulverized using mortar and pestle cooled with liquid nitrogen. The cartilage powder was weighed and proteins extracted in RIPA buffer (1 g of tissue powder/3 ml extraction buffer) overnight at 4°C on a roller shaker. The protein extract was centrifuged at 11,000 *g*, at 4°C for 30 min and the supernatant containing the solubilized proteins was transferred to a new tube. Centrifugation was repeated until the supernatant appeared clear.

Proteins were quantified using the BCA assay, with Pierce BCA Protein Assay Kit (Life Technology), following the manufacturer’s instructions. Protein extracts (20 μg/lane) were denatured and reduced by boiling for 10 min at 99°C in Loading Buffer (NuPAGE^®^ LDS Sample Buffer, Thermo Fisher Scientific), supplemented with 10% β-mercaptoethanol and loaded onto 10% sodium-dodecyl-sulfate polyacrylamide gel electrophoresis (SDS-PAGE) gels. A protein ladder (Precision Plus Protein^TM^ Dual Colour Standards, #1610374, Bio-Rad) was run alongside the samples for molecular weight estimation. Proteins from the gel were transferred onto a nitrocellulose membrane (Hybond-C Extra, GE Healthcare, Life Sciences, Amersham, United States) at 15 V overnight at 4°C, then blocked with 5% milk powder in 0.5% Tween 20 in TBS (TBS-T) for 1 at room temperature, and incubated with an anti-Collagen 2 rabbit polyclonal Ab (Supplementary Table 3) overnight at 4°C. Membranes were washed for 3 × 10 min with TBS-T, incubated with the horseradish peroxidase (HRP)-conjugated polyclonal goat anti-rabbit IgG for 1 h at room temperature and washed again. The bound antibody was detected using ECL Western Blotting reagents (GE Healthcare, Life Science) following the manufacturer’s instructions.

### Histological Analysis

Tissue and spheroid sections were stained for hematoxylin and eosin (H&E), Alcian Blue (glycosaminoglycans), Alcian Blue/Periodic Acid – Schiff (acidic and neutral mucins) and Alizarin Red (calcium deposition) using standard histological methods. Stained sections were imaged using the Zeiss Axioplan 2IE Mot Microscope System (Carl Zeiss, Jena, Germany) and the Axiovision software (Carl Zeiss). Cell nuclei and vessel counts were performed with ImageJ/Fiji, using the automatic Particle Analysis tool or the manual Cell Counter plugin, considering only those found in the chondrium layer. For each tissue, cell nuclei were counted in three independent fields, in at least 4 sections; for vessel quantification, at least 12 sections per tissue were imaged. The average numbers of cell nuclei and vessels in each tissue was expressed per unit area. Phase contrast pictures of tissues were taken using an inverted microscope Olympus IX71 equipped ORCA-R2 digital camera (Hamamatsu Corp., Bridgewater, NJ, United States). Differential interference contrast (DIC) pictures of spheroid sections were acquired using the Zeiss Axioplan 2IE Mot Microscope System (Carl Zeiss).

### Immunofluorescence

Cells or spheroid sections were fixed in 4% PFA prior to immunofluorescence protein detection. After incubation for 30 min with a blocking/permeabilizing buffer (10% FBS, 3% BSA, and 0.2% Triton X-100 in PBS), samples were incubated with primary antibodies for 2 h at room temperature and then for 1 h with fluorescent dye-conjugated secondary antibodies and Hoechst 33258 (2 μg/ml) at room temperature. All antibodies used are listed in Supplementary Table 3. Negative controls were incubated with the secondary antibody only. When tissue sections were permeabilized, 1% Triton X-100 was supplemented to the blocking buffer, and samples were incubated for 1 h. When sections were stained with anti-Collagen 1, -Collagen 2 and -Elastin (ELN) antibodies, samples were pre-treated with 0.1% trypsin for 20 min at 37°C, followed by washes in deionized H_2_O and PBS. Images were acquired either using an inverted microscope Olympus IX71 equipped ORCA-R2 digital camera (Hamamatsu Corp., Bridgewater, NJ, United States) or by confocal laser scanning microscopy (LSM 710, Carl Zeiss, Jena, Germany). Image analysis was performed using Fiji/ImageJ software.

### Horseradish Peroxidase (HRP) Staining

Horseradish peroxidase staining was performed using the Vectastain Elite ABC kits (Vector Laboratories, Burlingame, CA, United States), according to manufacturer’s protocols. All antibodies used are listed in Supplementary Table 3. Antigen retrieval was performed via trypsin digestion (0.1% trypsin for 20 min at 37°C, followed by washes in deionized H_2_O and PBS). Primary antibody was incubated overnight at 4°C, followed by incubation in 0.3% hydrogen peroxide in methanol for 30 min at room temperature and 3 × 10 min washes in 1× PBS, to inactivate endogenous peroxidases. A 1 h biotin-conjugated secondary antibodies incubation, at room temperature was followed by a 30 min incubation with ABC mixture solution containing biotinylated HRP (Vectastain Elite ABC kits, Vector Laboratories, Burlingame, CA, United States) at room temperature, and finally the color was developed using DAB (3,3′-diaminobenzidine) solution (DAB Peroxidase substrate, Vector Laboratories). Stained sections were dehydrated through ascending ethanol solutions, mounted using DPX Mounting Medium (Thermo Fisher Scientific), and imaged using the Zeiss Axioplan 2IE Mot Microscope System (Carl Zeiss) and the Axiovision software (Carl Zeiss).

### Statistical Analysis

Data are expressed as mean ± standard error of the mean (S.E.M). Statistical analysis was performed using GraphPad Prism 7 (GraphPad software Inc., La Jolla, CA, United States). Each experiment was performed on cells or tissues from one to five patients (N), and replicates in each experimental group were *n* ≥ 3. Statistical significance was evaluated by Mann–Whitney test or Kruskal–Wallis, followed by a Dunn’s *post hoc* test. A *p* value equal to or less than 0.05 was considered as statistically significant.

## Results

### Comparison of Native Human Normal and Microtic Ear Cartilages

**FIGURE 1:**
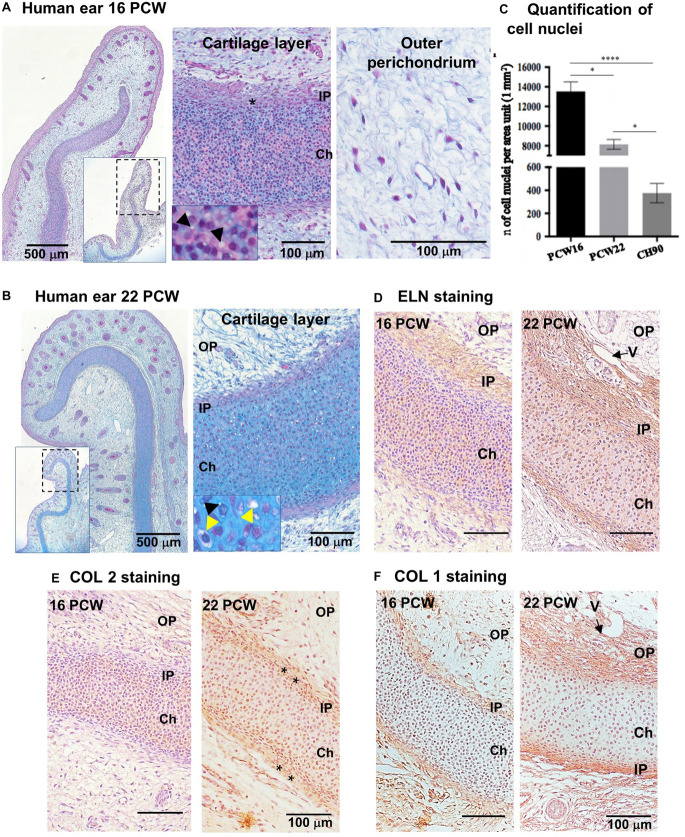
Characterization of human developing normal ear cartilage. Morphology and expression of ELN, COL2, and COL1 in 16 and 22 post-conception weeks (PCW) human ear cartilage sections. **(A,B)** Alcian Blue/Periodic Acid – Schiff (PAS) staining sections showing the developing ear and high magnification of the cartilage layer (chondrium) with developing chondroblasts (black arrowheads = peri-cellular cavities) ad chondrocytes (yellow arrowhead = lacunar space), and the surrounding outer perichondium (OP), at 16 **(A)** and 22 **(B)** PCW. Insets show lower magnification pictures of the ear section, and dashed rectangles indicate the field enlarged in the correspondent pictures. Asterisks indicate thick and irregular, developing inner perichondrium (IP). **(C)** Quantification of cell nuclei in the chondrium layer of fetal (PCW16 and PCW22) and pediatric ear normal cartilage sections (*n* = 4, in which at least three independent fields were analyzed). Data are expressed as mean ± SEM. Significance of differences (Kruskal–Wallis test and Dunn’s *post hoc* test, confidence level = 95%): ^∗^*p* < 0.05 and ^****^p < 0.0001; *n* = number. **(D)** Elastin, **(E)** COL2, and **(F)** COL1 expression by HRP immunohistochemistry shown by brown staining, in 16 and 22 PCW. Nuclei counterstained by hematoxylin. V = vessel.

Microtic ear cartilages were compared with developing normal ear and pediatric auricular cartilage to assess whether microtic cartilages display an immature phenotype. Developing human fetal external ears at 16 and 22 PCW (*N* = 1) were analyzed by histology and immunohistochemistry on paraffin sections (Figure 1). At both 16 and 22 PCW, two zones were identified in the developing cartilage layer: (i) a central layer, highly cellularized, containing immature chondroblasts with round nuclei and a few small peri-cellular cavities, identifiable as the chondrium, and (ii) the inner perichondrium, a thin, regular layer which contained spindle-like/fibroblastic shaped cells (we define these cells as CSPCs, according to Kobayashi et al., 2011a; Jiang and Tuan, 2015; Zhang et al., 2017). In many areas of the 16 PCW ear cartilage, the inner perichondrium appeared irregular (Figure 1A). In the 22 PCW ear, peri-cellular cavities and a few chondrocytes embedded in lacunae were apparent in the cartilage layer at high magnification (Figure 1B). Cells were sparser in the developing cartilage layer of 22 PCW ear sections compared to 16 PCW, as demonstrated by quantification of cell nuclei per unit area in tissue sections (Figure 1C). At 22 PCW, the cartilage layer was surrounded by an abundant OP containing fibers, small blood vessels, and sparse cells. Elastic cartilage proteins such as ELN and Collagen 2 (COL2) were already expressed in the inner perichondrium and developing cartilage by 16 PCW, but increased accumulation of COL2 in the inner perichondrium, where CSPCs are located, was observed at 22 PCW. Collagen 1 (COL1) reactivity was detected in the inner and OP, but not in the developing chondrium layer, both at 16 and 22 PCW (Figures 1D–F and Supplementary Figures 1A,B, for negative controls).

**FIGURE 2:**
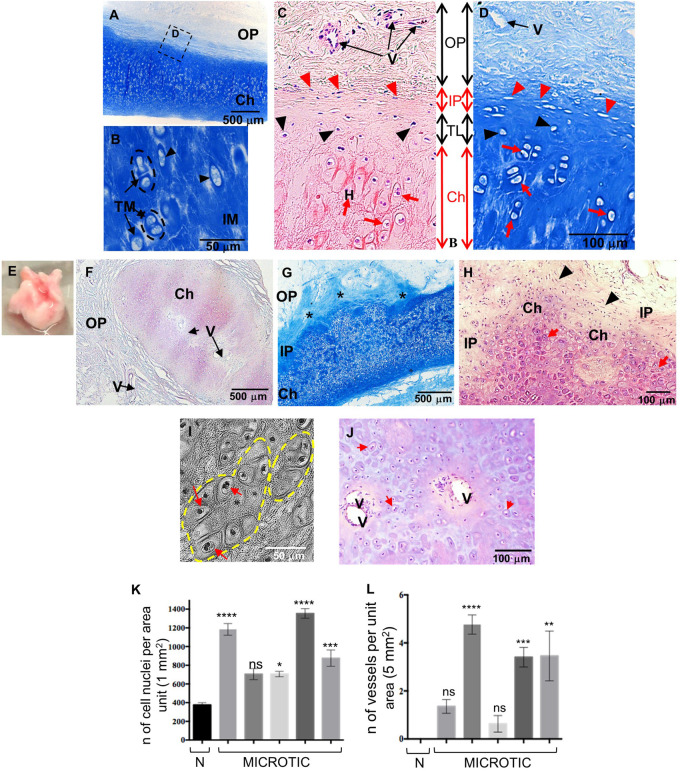
Histology of human normal and microtic pediatric ear cartilage. **(A–D)** Sections of normal auricular cartilage from a 9-year-old individual stained with Alcian Blue **(A,B,D)** or H&E **(C)**. Note that the chondrium layer (Ch) enlarged in **(B)** contains chondrocytes in lacunae (black arrowheads) surrounded by territorial matrix (TM), and small isogenous groups (black dashed circles). **(C,D)** Multi-layered tissue architecture. Outer perichondrium containing fibers and vessels (V); inner perichondrium containing cartilage stem/progenitor cells (CSPCs, red arrowheads); transitional layer containing chondroblasts (black arrowheads); mature chondrium containing chondrocytes in lacunae (red arrows). **(E)** Example of a biopsy of microtic cartilage. **(F–J)** Representative sections of microtic auricular cartilage from a 9-year-old individual stained with H&E **(F,H,J)** or Alcian Blue **(G)**. **(F)** Amorphic islet of cartilage containing vessels (V) in the chondrium (also shown in **I**) and surrounded by outer perichondrium. **(G)** Cartilage multi-layered architecture is disrupted, and IP is irregular (asterisks) in many areas. **(H)** At high magnification chondrium (with round cells) and IP (with CSPCs) are found in the same layer. **(I)** Phase contrast image of big isogenous groups (yellow dashed circles). **(J)** Vessels in the chondrium layer of microtic ear cartilage. Red arrows, chondrocytes in lacunae; IP, inner perichondrium; OP, outer perichondrium; TM, territorial matrix; IM, interterritorial matrix. **(K)** Quantification of cell nuclei in the chondrium layer of normal (*N* = 1) and microtic ear cartilage patients (*N* = 5). **(L)** Quantification of vessels in the chondrium layer of normal and microtic ear cartilage patients. Data are expressed as mean ± SEM. Significance of differences (Kruskal–Wallis test and Dunn’s *post hoc* test, confidence level = 95%): *p < 0.05, **p < 0.01, ***p < 0.001, and ****p < 0.0001 vs normal ear. *n* = number; ns = non-significant. *N* = normal.

By 9 year of age, the normal external ear cartilage, was hard and elastic with a typical white and translucent appearance. Histological examination by H&E and Alcian Blue staining of this biopsy showed a thin and regular layer of cartilage tissue with a high GAG content, as indicated by the staining intensity, surrounded by a fibrous OP (Figures 2A,B,D). As evident at high magnification, normal elastic cartilage presented a multi-layered architecture including: (i) the OP, consisting of connective fibers and vessels, (ii) the inner perichondrium, a thin, regular layer containing CSPCs (spindle-like cells), (iii) the transitional layer, containing chondroblasts, and (iv) the chondrium or mature cartilage layer, containing chondrocytes (Figures 2C,D). Chondrocytes were in lacunae and organized in vertical columns (Figures 2B–D). Several isogenous groups were identified, each containing two–three chondrocytes (Figure 2B).

**FIGURE 3:**
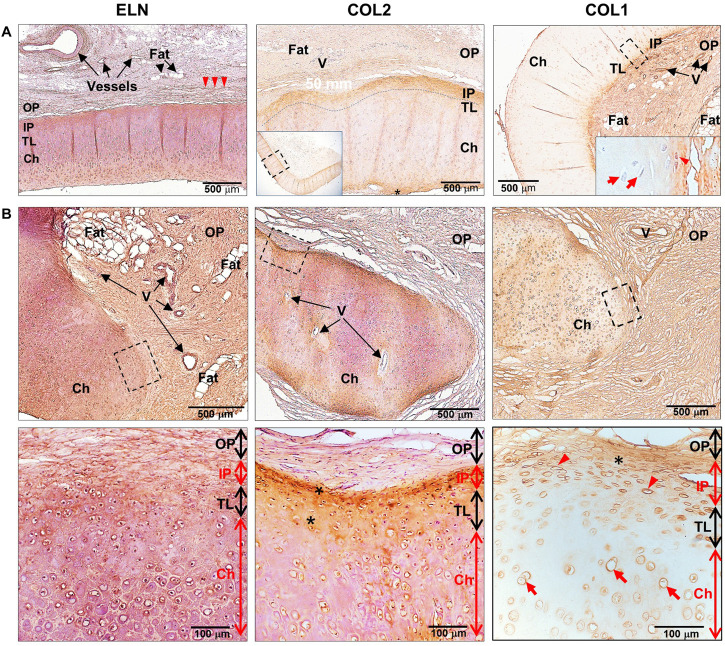
Expression of extracellular matrix proteins in human healthy and microtic ear cartilage. ELN, COL2, and COL1 expression in **(A)** human pediatric normal (*N* = 1) and (B) microtic (*N* = 2, *N* = 4, and *N* = 4, respectively) ear cartilage, by HRP immunohistochemistry shown by brown staining. Nuclei counterstained by hematoxylin. **(A)** COL2 panel: the inset shows the tissue section at low magnification, and the dashed rectangle indicates the area enlarged in the panel; COL1 panel: the inset shows the enlarged area indicated by the dashed rectangle. **(B)** The dashed rectangles in the top panels indicate enlarged areas shown in the bottom panels. For each tissue, at least three sections were examined, in which at least four independent regions were analyzed. Red arrowheads, cartilage stem/progenitor cells (CSPCs); red arrows, chondrocytes; arrowheads, elastin fibers in the outer perichondrium; OP, outer perichondrium; IP, inner perichondrium; TL, transitional layer; Ch, developing chondrium; V, vessels.

In contrast to normal ears, microtic cartilages (*N* = 5) were amorphic with high variability in shape and size observed between patients. An example of the gross anatomy of microtic cartilage is shown in Figure 2E. Histological analysis showed that although microtic cartilages contained chondrocytes in lacunae that produced GAGs, there was major disruption of the structural organization typical of normal ears (Figures 2F–I). A key feature of microtic cartilage was the presence of unshaped islets of cartilage surrounded by abundant fibrous connective tissue and fat instead of a regular, thin layer of elastic cartilage (Figure 2F). In many areas the multi-layered architecture of cartilage was missing, and the inner perichondrium and chondrium appeared very irregular compared to normal elastic cartilage (Figures 2G,H). Furthermore, microtic ear cartilage appeared hyper-cellular compared to normal ear cartilage (Figures 2G,J,K), and chondrocytes were not organized in vertical columns as in normal cartilage (Figures 2H,I,J). The interterritorial matrix was present only in some areas and in small patches, whereas it was completely absent in other parts of the chondrium. Very large isogenous groups, including up to 10–12 chondrocytes, were found in the chondrium of microtic cartilage (Figure 2I). Finally, small vessels were found in the chondrium layer of microtic ear cartilage, in addition to the OP, but not in the chondrium of normal ear cartilage (Figures 2J,L, 3A).

Analysis of ECM proteins, ELN and COL2, in pediatric normal and microtic cartilages showed comparable expression patterns (Figure 3 and Supplementary Figures 1C–F, for negative controls). In normal ear cartilage, ELN was detected in the inner perichondrium, translational layer and mature chondrium layer, and elastic fibers were also present in the OP. Similarly, in microtic ear cartilage, ELN was expressed in both amorphic cartilage islets and the surrounding OP. COL2 was found in the inner perichondrium and translational layer, where CSPCs and chondroblasts were present, both in normal and microtic ear cartilage. In contrast, COL1 expression pattern differed in microtic and normal ear cartilages. While in normal cartilage COL1 expression was restricted to the inner and OP, in microtic ear cartilage it was found also within the amorphic cartilage islets.

### Characterization and Differentiation Potential of Human Pediatric Precursor Cells From Normal and Microtic Ear Cartilage and Adipose Tissue in 2-Dimensional (2D) Cultures

**FIGURE 4:**
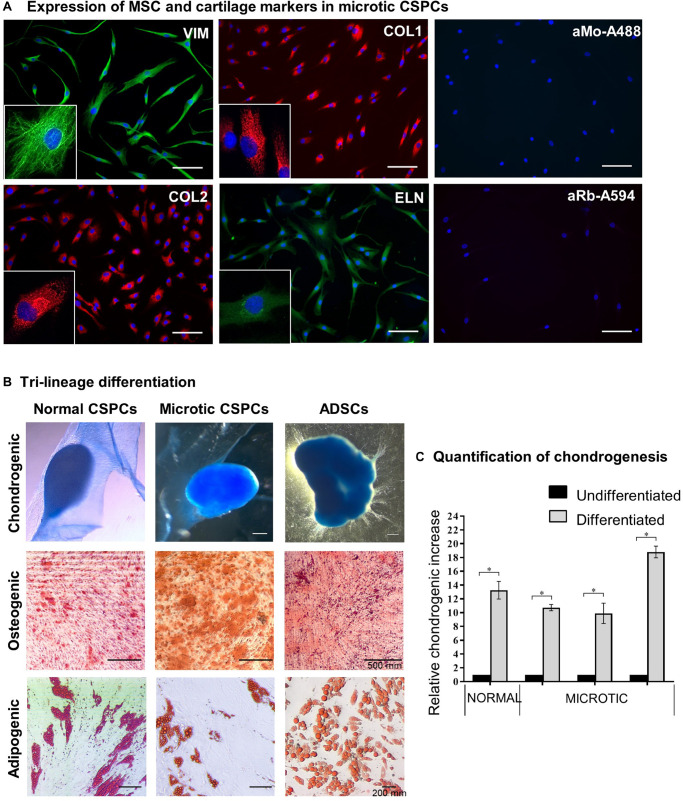
Characterization of microtic cartilage stem progenitor cells. **(A)** Immunofluorescence staining of mesenchymal stem cell (Nestin, NES; Vimentin, VIM; Collagen 1, COL1) and cartilage-associated proteins (Collagen 2, COL2; Elastin, ELN) in microtic CSPCs (cartilage stem/precursor cells). aMo-A488 (Anti-mouse Alexa Fluor^®^ 488) and aRa-594 (Anti-rabbit Alexa Fluor^®^ 594) are negative controls of staining, in which the cells were incubated with secondary Abs only. Scale bars = 100 μm. **(B)** Normal CSPCs (*N* = 1), microtic CSPCs (*N* = 3), and ADSCs (*N* = 3) stained with Oil Red O (adipogenic), Alcian Blue (chondrogenic) and Alizarin Red (osteogenic) after 3 weeks in differentiation media. All pictures are representative of at least *n* = 3 biological replicates, in which four independent fields were examined. **(C)** Quantification of chondrogenesis in normal (*N* = 1) and microtic (*N* = 3) CSPCs, cultured for 3 weeks in either proliferation (undifferentiated) or chondrogenic medium (differentiated), based on Alcian Blue measurement at OD_595_. Data are expressed as fold difference, taking undifferentiated control as 1; data are mean (*n* = 4) ± SEM Mann–Whitney test, *p < 0.05.

The behavior and differentiation potential of cells derived from either normal or microtic cartilages was investigated in 2D cultures. Cells from both microtic and healthy ear cartilage explants migrated out within 5–7 days after plating and displayed the same fibroblast-like morphology (spindle-shaped cells) with multiple, long protrusions (Figure 4A and Supplementary Figure 2). Based on their morphology and properties shown below, we will refer to these cells as CSPCs. In monolayer cultures, these cells expressed typical MSCs markers such as Vimentin (VIM) and COL1 as well as cartilage-associated proteins, such as COL2 and ELN, as assessed by immunofluorescence. COL1 and COL2 staining was intracellular and punctuated, and mainly localized around the cell nuclei. This is likely to reflect accumulation of procollagens in the endoplasmic reticulum and Golgi network in undifferentiated cells, as reported in previous studies (Bonfanti et al., 1998; Unlu et al., 2014). A similar pattern of expression was observed in ADSC primary cultures (Supplementary Figure 2), which were used for comparison. COL2 protein expression in microtic ear cells and ADSCs was also confirmed by Western Blotting (Supplementary Figure 3). Expression of *VIM*, *COL1*, and *ELN* in microtic and normal ear CSPCs and in ADSCs was detected also at the transcriptional level (Supplementary Figure 4). In contrast, the *COL2* (71 and 125 bp) transcript was barely detectable in the CSPCs. This could be explained by the long half-live and very slow turnover of ECM proteins. Therefore, these proteins can be still detected by immunohystochemistry when gene expression has been already down-regulated (Verzijl et al., 2000; Decaris et al., 2014). The cartilage-specific transcript Aggrecan (*ACAN*) was clearly detected in all cell lines, except for one microtic ear- and one ADSC line where it was barely detectable. Both CSPCs and ADSCs expressed Nestin (*NES*) gene, usually considered a marker of neural stem cells, but more recently found expressed also by pluripotent MSCs (Mendez-Ferrer et al., 2010). Other chondrogenesis markers, such as SRY-Box Transcription Factor 9 (*SOX9*), Runt-related transcription factor 2 (*RUNX2*), and collagen 10 (*COL10*) were detected in all cell lines tested, but *COL10* was not observed in ear cartilage, as expected in non-hypertrophic tissue (Supplementary Figure 4).

Normal and microtic CSPCs were cultured in 2D in adipogenic, chondrogenic or osteogenic media to assess their differentiation potential along three-mesenchymal lineages. ADSCs were used as positive control. Tri-lineage differentiation was observed in all CSPCs, as demonstrated by Oil Red O (lipid droplets: adipogenic), Alcian Blue (GAGs, glycosaminoglycans: chondrogenic) and Alizarin Red (calcium deposits: osteogenic) staining, similarly to ADSCs (Figure 4B). When grown in 2D in chondrogenic differentiation media, CSPCs condensed and spontaneously formed 3D aggregates by 10–15 days of culture. Undifferentiated cultures grew as monolayers and never aggregated (data not shown). Quantification of GAGs produced upon chondrogenic differentiation was assessed in microtic and normal CSPCs (Figure 4C), and ADSCs (Supplementary Figure 5), by semiquantitative analysis of Alcian Blue staining. After 3 weeks of differentiation, GAG levels in differentiated ear-derived cultures were all increased compared to undifferentiated (^∗^*p* < 0.05 and were found around 3.5 times higher than in chondrogenic ADSCs).

### Characterization of Chondrogenically Differentiated Ear CSPC and ADSC Spheroids

**FIGURE 5:**
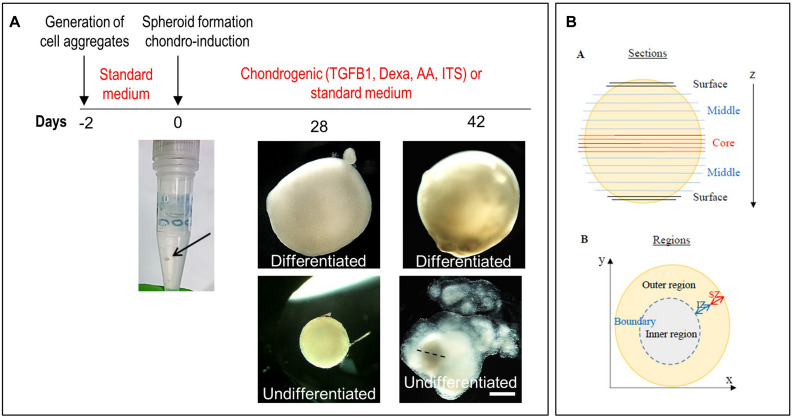
Generation of spheroids and induction of chondrogenic differentiation. **(A)** Timeline of the differentiation of ear CSPCs (cartilage stem/progenitor cells) into cartilage spheroids. The arrows at day 0 points at the cell aggregate after formation. Gross pictures show normal CSPCs cultured for 6 weeks in either standard (undifferentiated) or chondrogenic (differentiated) medium, imaged at 28 and 42 days of culture. The dashed line in 6 weeks undifferentiated spheroid indicates the core, considered to measure the diameter width. Pictures are representative of four undifferentiated/differentiated spheroids (*N* = 1). Scale bar = 500 μm. **(B)** Schematic representation of the spheroids. Spheroid sections are defined as surface, middle and core sections in relation to their position on the *z* axes. Different areas were identified in differentiate spheroid sections, referred to as: (i) inner region: area located in the inner part; (ii) outer region: area from the external surface to the core; and (iii) boundary: interface between the inner and outer region. In some samples, within the outer region two zones were identified, referred to as internal (IZ) and superficial zone (SZ).

We initially used normal CSPCs to set up a high-density spheroid culture system (Figure 5A), and then used this model to investigate the response of microtic CSPCs to a 3D environment. ADSCs were used for comparison. For simplicity we will refer to the spheroids generated using normal and microtic ear CSPCs and ADSCs, as normal and microtic spheroids, and ADSC spheroids, respectively. After 4 weeks in culture, both undifferentiated and differentiated normal spheroids showed a round shape and ivory-white color. Chondrogenically differentiated spheroids were around 2.5-fold bigger (mean diameter of differentiated/mean diameter of undifferentiated, *n* = 3, ^∗^*p* < 0.05), compared to the undifferentiated ones (Supplementary Table 4). Undifferentiated spheroids maintained a round shape up to the fourth week in culture. However, when maintained in culture for up to 6 weeks, they gradually lost contours and became very fragmented, with a small central core surrounded by loose matrix (Figure 5A). In contrast, differentiated spheroids maintained a well-defined, round shape and a firm consistency, and they were 3.3-fold bigger (mean diameter of differentiated/mean diameter of undifferentiated, *n* = 4, *p* < 0.05) than the undifferentiated ones (Supplementary Table 4).

**FIGURE 6:**
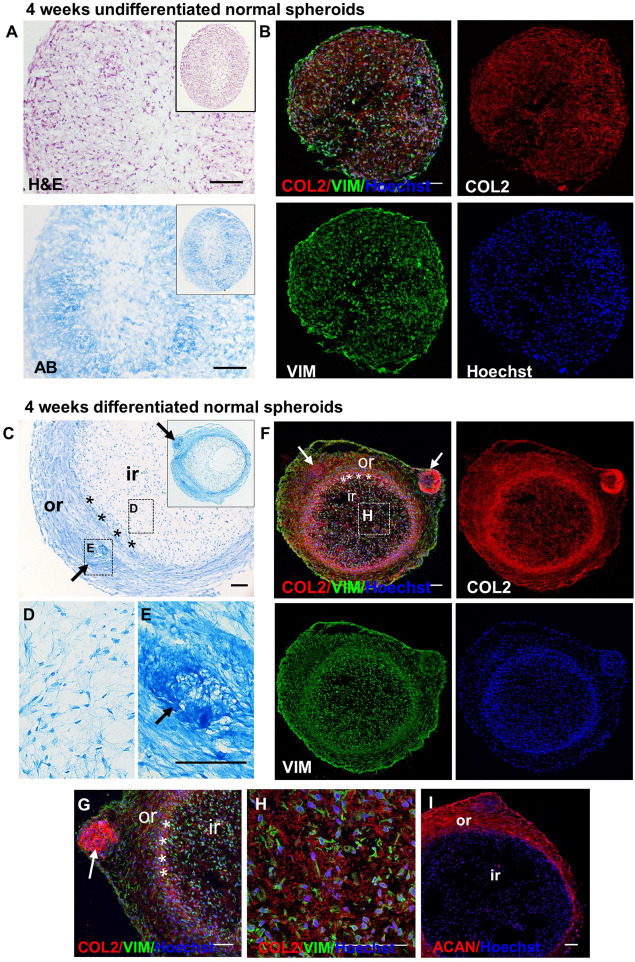
Detection of GAGs, chondrogenic and MSC markers in normal spheroids after 4 weeks of chondrogenic differentiation. Histological and immunofluorescence staining in core section from 4 week undifferentiated **(A,B)** and differentiated **(C–I)** normal CSPC spheroids. Sections were stained for Collagen 2 (COL2), Elastin (ELN), Vimentin (VIM) and Aggrecan (ACAN). Nuclei counterstained with Hoechst (blue). **(A,B)** Undifferentiated spheroid core sections stained by Hematoxylin and Eosin (H&E), Alcian Blue (AB) and **(B)** fluorescent antibodies. **(C–E)** Differentiated spheroid core sections stained by AB. Note the presence of a self-organizing structure, with a ring-like structure (outer region, or) and an inner region (ir), separated by a boundary region (asterisks), in the differentiated samples, but not in the undifferentiated controls. **(F–I)** Differentiated spheroid core sections stained by fluorescent antibodies. The insets in **(A)** and **(C)** show the low magnifications of the same section shown in the correspondent figure. The dashed rectangles in **(C)** and **(F)** show the areas enlarged in panels **(D,E,H)**. Arrows = cartilage nodules. Pictures are representative of *n* spheroids = 4, in which at least six sections were examined (*N* = 1). All scale bars = 100 μm.

**FIGURE 7:**
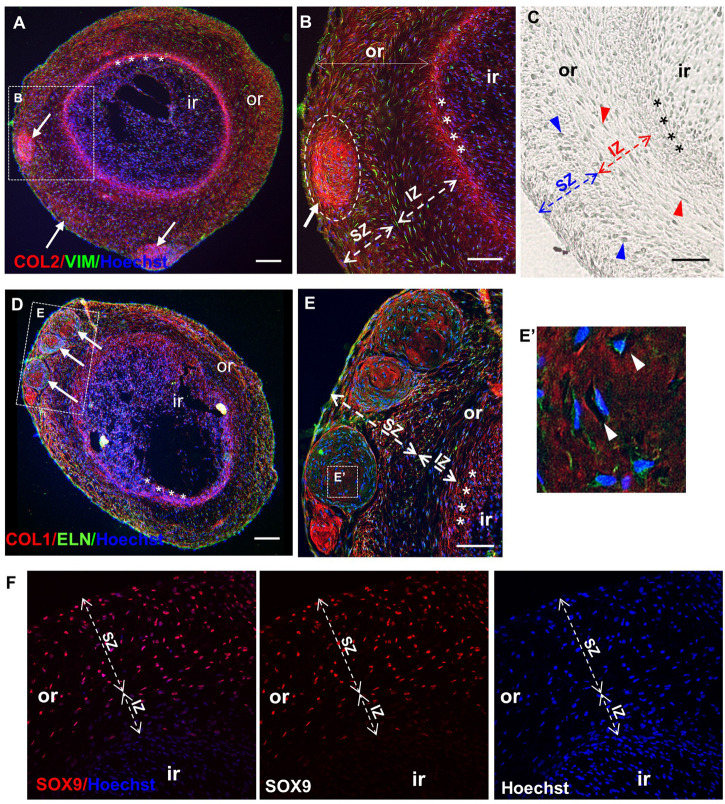
Maturation of normal spheroids after 6 weeks of chondrogenic differentiation. Immunofluorescence **(A,B,D–F)** and differential interference contrast (DIC) **(C)** pictures of core sections from normal CSPC spheroids, after 6 weeks in chondrogenic medium. Sections are stained for Collagen 2 (COL 2), Vimentin (VIM), Elastin (ELN), Collagen 1 (COL 1), and SRY-Box 9 (SOX 9). Nuclei are counterstained with Hoechst dye (blue). **(A,D)** Differentiated spheroids at low magnification show typical self-organizing structure, with an inner (ir) and outer region (or, dashed lines), where cartilage nodules (white arrows) are present, and a definite boundary (asterisks) between the inner (ir) and the outer region (or) (also shown enlarged in **B**). **(C,E)** Note the presence of two zones within the outer region in 6 weeks differentiated spheroids: a more superficial zone (SZ) containing round cells (blue arrowheads), and a more internal zone (IZ) located next to the boundary, containing spindle-like cells (red arrowheads). **(E,E’)** Cartilage nodules in the SZ containing developing chondrocytes in lacunae (white arrowheads in **E’**). **(F)** Staining for SOX9 was detected only in the outer region, but not in the internal region. Representative sections from the analysis of three spheroids where ≥6 sections and four independent fields/section were examined (*N* = 1). All scale bars = 100 μm.

Undifferentiated and chondrogenically differentiated normal spheroids were cryosectioned and analyzed by histological and immunofluorescence staining. The diagram in Figure 5B shows a schematic view of the spheroid and indicates the terms used for the analysis of the spheroid sections. Undifferentiated and differentiated normal spheroid cryosections displayed a very different morphology. In 4 weeks, undifferentiated spheroid sections stained with H&E and Alcian Blue, cells appeared homogeneously distributed both at the surface and in the core (Supplementary Figure 6A and Figure 6A); they produced GAGs and expressed COL2 and VIM throughout (Figure 6B). In contrast, the 4 weeks differentiated normal spheroids contained different regions with diverse cellular and matrix organization (Figure 6C). On the spheroid surface, cells appeared to have formed a tight and homogenous layer (Supplementary Figure 6B). In contrast, sections in the middle (Supplementary Figure 6C) and in the core (Figures 6C–I) of the spheroids contained distinct regions: (i) a spherical inner region with a low GAG content (Figures 6C,D), surrounded by (ii) a ring-like outer region, with a high GAG content as indicated by the strong AB staining (Figures 6C,E) separated by (iii) a boundary zone (Figures 6C,F,G). Cell density was significantly lower in the outer region than in the inner region (Supplementary Figure 8D). The external ring contained some cartilage nodules which stained intensely for Alcian Blue and COL2 (arrows in Figures 6C,E–G). Cells that formed the boundary zone were strongly COL2-positive (asterisks in Figures 6C,F,G). ACAN staining was detected only within the outer region (Figure 6I). In 6 weeks differentiated normal spheroids (Figure 7), these features appeared even more pronounced. We observed a more clearly defined boundary at the interface between the inner and outer regions (asterisks in Figures 7A,B), and the presence of two zones within the outer region, indicated as internal zone (IZ) and SZ, containing spindle-like and round cells, respectively (Figure 7C). Interestingly, (iii) cartilage nodules were only found within the SZ (Figures 7B,D,E); they were more numerous and more mature compared to those present in 4 weeks differentiated spheroids, as they contained developing chondrocytes in lacunae (Figure 7E’). In some regions of big nodules and in smaller nodules, COL1 expression was detected (Figure 7E). Finally, the protein transcribed by the master regulator gene of chondrogenesis, *SOX9*, was expressed only by cells in the external ring of the spheroid, but not in the inner region, both in 4 weeks (Supplementary Figure 7) and 6 weeks differentiated normal spheroids (Figure 7F).

**FIGURE 8:**
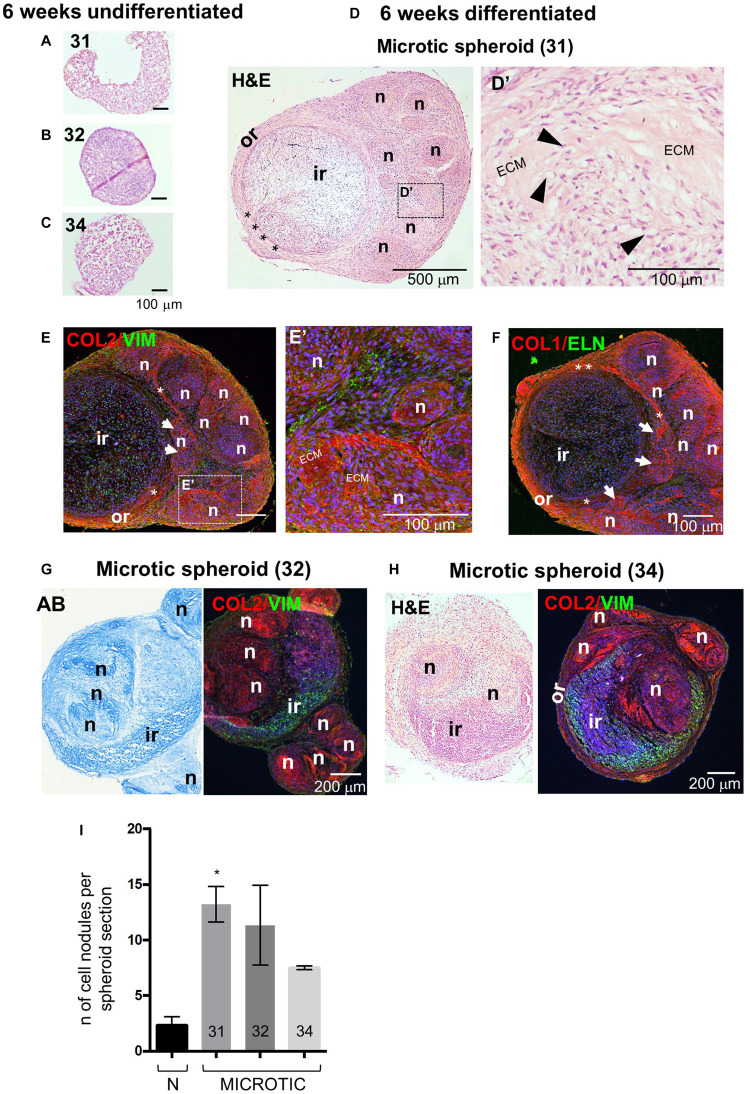
Histology, protein expression and nodule formation in microtic spheroids after 6 weeks of chondrogenic differentiation. **(A–C)** Hematoxylin and Eosin (H&E) staining in core section from microtic spheroids from cartilage stem/progenitor cell (CSPC) lines CSPC-31 **(A)**, CSPC-32 **(B)**, and CSPC-34 **(C)**, cultured for 6 weeks in standard proliferation (Undifferentiated) medium. **(D–H)** Sections from microtic spheroids cultured for 6 weeks in chondrogenic (Differentiated) medium. Sections are stained for Collagen 2 (COL 2), Vimentin (VIM), Elastin (ELN), and Collagen 1 (COL 1). Nuclei are counterstained with Hoechst dye (blue). **(D–F)** Microtic spheroids, line CSPC-31. Hematoxylin and Eosin (H&E) **(D,D’)** and immunofluorescent **(E,E’,F)** staining in middle **(D,D’)** and core **(E,E’,F)** sections, showing the **(D–F)** stereotyped structure of CSPC-31-differentiated spheroids. Or, outer region; Ir, inner region; asterisks, boundary; *n*, cartilage nodules; white arrows in **(E,F)**, cartilage nodules close to the ir. **(D’,E’)** High magnification pictures show cartilaginous nodules, highly cellularized, containing patches of deposited extracellular matrix (ECM), and chondrocytes in lacunae (black arrowheads). **(G)** Alcian Blue (AB) and immunofluorescent pictures of core sections from microtic ear-derived cell spheroids, line CSPC-32. **(H)** H&E and immunofluorescent pictures of core sections from microtic ear-derived cell spheroids, line CSPC-34. **(I)** Quantification of cartilage nodules in normal and microtic spheroid sections from three lines (31, 32, and 34). Data represent analysis of three spheroids per line, apart from line 34 (two spheroids) with nodules counted in three sections/spheroid in three independent fields/section. Data are expressed as mean ± SEM. Significance of differences (Kruskal–Wallis test and Dunn’s *post hoc* test, confidence level = 95%): *p < 0.05.

The spheroid culture system was then used to study the behavior of microtic spheroids and assess whether this was consistent across patients. To this purpose we compared microtic spheroids from three independent patients (31, 32, and 34 of Supplementary Table 1). As the more mature structure was observed in normal spheroids after 6 weeks compared to 4 weeks in culture, we focused on the later time point for the analysis of microtic spheroids. After 6 weeks, all undifferentiated microtic spheroids were very fragile, and much smaller than the differentiated samples (Figures 8A–C). In some undifferentiated samples the cells did not form a sphere but remained as discoidal pellets (Figure 8A). In contrast, all microtic spheroids maintained in chondrogenic medium had a white color, a glossy and translucent appearance, and a firm texture, similar to differentiated normal spheroids (data not shown). However, they displayed irregular shapes rather than being spherical. Various morphologies were observed in differentiated microtic spheroid sections (Figures 8D–H). Spheroids from all lines contained numerous cartilage nodules (Figures 8D–H) and discrete areas of ECM deposition (Figures 8D’,E’), as demonstrated by histological analysis and COL2 staining. Differentiated spheroids from two microtic CSPC lines (32 and 34, Figures 8G,H) presented a very different morphology from normal spheroids, with no well-defined ring-like outer region or boundary zone, and cartilage nodules present within the inner-like region. Although spheroids from the third microtic line investigated, 31, had an identifiable circular inner region and outer region like control spheroids (Figure 8D), they contained a higher number of cartilage nodules than controls (Figure 8I) very close to the inner region (Figures 8E,F), and the boundary was discontinuous (Supplementary Table 5), with a lack of definition within the outer zone between IZ and SZ (Figures 8D,F). No evidence of endochondral ossification was observed in the differentiated CSPC spheroids, as indicated by negative staining for alkaline phosphatase and lack of calcium deposition (Supplementary Figures 8A–C).

Adipose-tissue derived stem cells chondrogenic spheroids were generated for comparison. They also showed a firm consistency and a round shape, with white color and a glossy appearance after 6 weeks differentiation (Supplementary Figure 8A). Multiple cartilage nodules were observed throughout the differentiated ADSC spheroids, both in surface and core sections, as demonstrated by both Alcian Blue staining and COL2 immunostaining (Supplementary Figures 8B,C). However, no stereotyped structure was observed in ADSC spheroid sections.

## Discussion

Here, we characterized the macroscopic and microscopic structure of microtic ear cartilage and systematically compared it to normal fetal and postnatal auricular cartilage, revealing previously undescribed features of the pathology. Furthermore, we compared CSPCs from human normal and microtic ears in 2D and 3D high density culture systems, called spheroids, for the first time. Assessment of microtic spheroids against normal developing auricular cartilage and postnatal microtic cartilage suggested that they recapitulate many characteristics of microtic ears, which were not apparent when microtic CSPCs were studied in 2D systems.

### Microtic Ear Cartilage Appears Underdeveloped *in vivo*

As shown here, the inner perichondrium in developing normal ear cartilage appears as an irregular layer at 16 PCW and has become thinner and more defined by 22 PCW, resembling more closely postnatal cartilage. Also, cell density changes with development, progressively decreasing, and in mature cartilage chondrocytes are interspersed within abundant and homogenous ECM. Notwithstanding some inter-patient variability and possibly different etiologies of the disease, there are remarkable similarities between human microtic cartilages as compared to normal cartilage. These include disruption of the typical multi-layered structural organization of elastic cartilage, presence of unshaped islets of cartilage, hyper-cellularity, expression of COL1 in the chondrium (as shown also by Dahl et al., 2011; Pappa et al., 2014) and, most surprisingly, the presence of blood vessels in the chondrium. Many of these features are consistent with the hypotheses that in microtia ear cartilage maturation are arrested at an intermediate stage, and neural crest cell patterning into the first and second pharyngeal arch during ear development is defective, as previously suggested (Luquetti et al., 2012). Infiltration of collagen fibers from the perichondrium into the lacunar cartilage of microtic ears has been briefly mentioned only in one study (Gu et al., 2018), whereas other studies reported an overall good preservation of the microtic cartilage structure (Dahl et al., 2011; Pappa et al., 2014). Discrepancies with previous studies may depend on the rather small number of patient samples they analyzed. Together, our analysis of microtic cartilage from five patients is more in agreement with observations by Gu et al. (2018), suggesting abnormalities in perichondrium and lacunae organization, and support the view that at the cellular level microtic cartilage is not simply a smaller normal cartilage, but a cartilage that is unable to reach the maturity and organization of healthy auricular cartilage.

### Chondrium Vascularization Is a Landmark of Microtic Ear Cartilage

A novel and unexpected finding that has emerged from our analysis of microtic ears is the presence of small blood vessels in the chondrium. To our knowledge, presence of blood vessels has never been reported in the chondrium of normal cartilage, neither in fetal, nor pediatric (this study), nor adult ear, consistent with the notion that cartilage is an avascular tissue (Chung and Burdick, 2008; Akkiraju and Nohe, 2015; Bernhard and Vunjak-Novakovic, 2016). The only cartilaginous tissue that becomes vascularized in a controlled physiological manner is the hypertrophic cartilage in the growth plates of long bones, ribs, and vertebrae during endochondral ossification (Hattori et al., 2010). Nevertheless, no sign of trabecular bone formation and mineralization were found in microtic cartilages or spheroids, neither in ours (data not shown) nor in previous studies. While at present we do not know what causes microtic cartilage to become vascularized, nor whether it is the cause or a consequence of the disease, we believe this is an important finding that can guide investigation into the pathology of microtia. For example, comparing the expression of vascular endothelial growth factor (*VEGF*), the master regulator gene of angiogenesis, and other cartilage-derived angiogenic and antiangiogenic molecules (such as the angiogenic cartilage-derived inhibitor, CDI; Moses et al., 1990), as well as endothelial cell stimulating angiogenic factor (ESAF) (Brown and Weiss, 1988) in microtic and normal ear tissues, may help to elucidate the molecular mechanisms of aberrant vascularization, and its potential role in the etiogenesis of the disease.

### Auricular CSPCs Are Akin to Mesenchymal Stem Cells and Achieve a Stereotyped Organization in 3D

We have shown that human microtic CSPCs can be easily isolated and expanded to large numbers as previously reported (Kobayashi et al., 2011a; Zhou et al., 2018). Our additional characterization in 2D cultures supports the view that auricular CSPCs behave like MSCs. In addition to expressing the typical MSC surface markers, CD73, CD90, and CD105 (Gardner et al., in preparation), they express intracellular markers typical of MSCs as well as chondrogenic/osteogenic lineage markers and display three-mesenchymal lineage differentiation capability. Similar results were obtained with CSPCs derived from normal ear cartilage.

Consistent with other studies, NES, VIM, COL1 and ELN were detected both at the mRNA and protein levels in CSPCs as well as in ADSCs (Schaffler and Buchler, 2007; Guasti et al., 2012; Guasti et al., 2014). Non-secreted COL2 protein was detected in undifferentiated cells, whereas expression of the transcript, previously demonstrated by RT-qPCR both in ADSCs and CSPCs, was below the sensitivity of RT-PCR. This is consistent with the long half-life of COL2 and suggests a very low turnover of the protein in the undifferentiated cells (Verzijl et al., 2000; Decaris et al., 2014; Guasti et al., 2014). This is the first report of COL2 expression also at the protein level in undifferentiated auricular CSPC and ADSC cultures and parallels a study where COL2 protein was detected in undifferentiated human umbilical cord derived-MSCs (Ahn et al., 2015).

All precursor cells analyzed in this study also expressed *SOX9*, *RUNX2*, and *ACAN* genes. Low level of *ACAN* mRNA in undifferentiated cultures was previously shown in ADSCs, and in human MSCs from either umbilical cord blood (UCB-MSCs) or bone-marrow (BM-MSCs) (Mwale et al., 2006a, b; Guasti et al., 2012; Gomez-Leduc et al., 2016). This is not surprising, as *ACAN* is detected in several tissues, including in the brain parenchyma, and not restricted to mature cartilage (Hering et al., 2020; Hermann et al., 2020; Mitlohner et al., 2020). Also, *RUNX2* expression was previously shown in undifferentiated hBM-MSCs (Mwale et al., 2006a). Together, results from ours and other studies suggest that MSCs are multipotent cells that express constitutively a broad set of genes, which are selectively upregulated or downregulated upon differentiation, depending on the stimuli provided in culture. Importantly, expression of a few genes in isolation is not sufficient to determine the extent of chondrogenic differentiation and needs to be combined with assessment at the morphological and protein level.

In 2D cultures microtic cell behavior upon induction of chondrogenic differentiation appears fairly similar to normal CSPCs. Undifferentiated 3D CSPC spheroids were also quite homogeneous and tended to disaggregate and fall apart over time. This is consistent with the only study on microtic spheroid cultures published so far, where spheroids were maintained in growth medium and not exposed to a chondrogenic medium (Zhang et al., 2014). In contrast, as shown here, when CSPC spheroids are induced to undergo chondrogenic differentiation, a more complex and stereotyped organization becomes apparent. Together, this highlights the self-organization capability of CSPCs upon chondrogenic induction and the importance of using more complex systems for human tissue modeling *in vitro*. To some extent, microtic CSPCs grown in 3D replicated the behavior observed in previous studies, where CSPCs could self-renew and reconstitute cartilage chondrium and perichondrium after *in vivo* grafting (Yanaga et al., 2009; Kobayashi et al., 2011a; Yanaga et al., 2012; Zhou et al., 2018).

### Microtic Ear Cell-Derived Spheroids Show Similar Features to Immature Developing Ear Cartilage and Microtic Ear Cartilage *in vivo*

**FIGURE 9:**
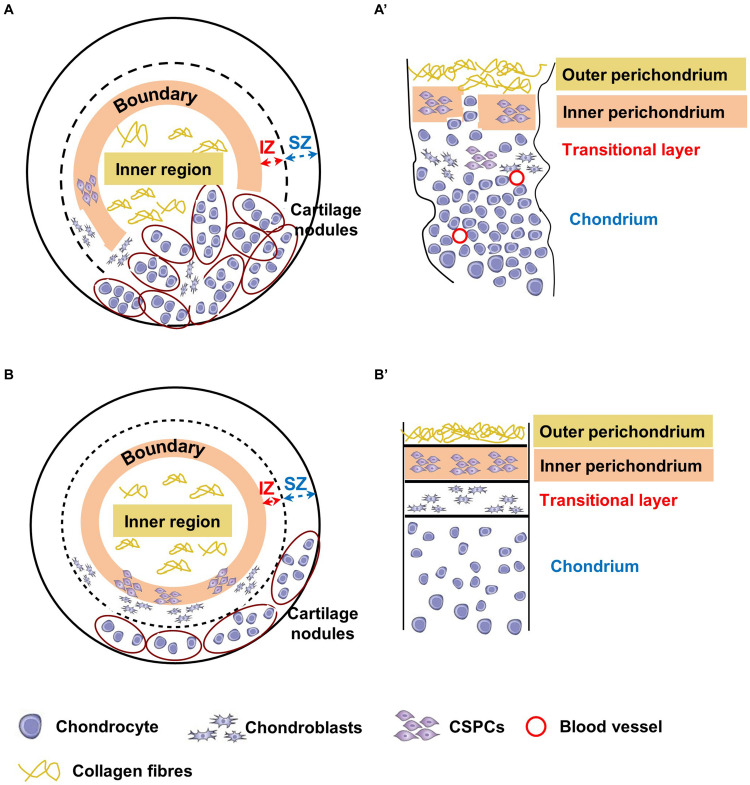
Schematic representation of chondrogenically differentiated spheroids and native cartilages from microtic and normal ears. Corresponding tissue layers in spheroids and native cartilages are indicated by the same color coding. The inner region of the spheroids **(A,B)** corresponds to the outer perichondrium in native tissues **(A’,B’)**, the boundary between the inner and outer region to the inner perichondrium, the internal zone (IZ) to the transitional layer, and the superficial zone (SZ) to the chondrium. **(A**,**A’)** *In microtic spheroids and microtic tissue, the boundary*/IZ and inner perichondrium/transitional layer, respectively, are discontinuous with mature chondrocytes present across these boundaries. The densely cellularized cartilage nodules accumulated to one side of the spheroid resemble amorphic islets of cartilage present in native microtic cartilage. **(B,B’)** *In normal spheroids, boundaries are maintained, with cartilage nodules restricted to the SZ, where they form a layer similar* to the chondrium of normal native ear cartilage.

Remarkably, microtic chondrogenic spheroids *in vitro* appear to recapitulate some abnormal features observed in the microtic cartilage *in vivo*. Although they can clearly undergo chondrogenic differentiation, the numerous cartilage nodules that accumulate to one side of the spheroids are highly cellularized and contain only small areas of ECM. Moreover, the cartilage nodules in microtic spheroids appear to be in close contact with the inner region, and the boundary between the inner and outer region was missing, as the inner perichondrium and transitional layer in microtic cartilage (Figure 9A).

Our observations of normal CSPCs chondrogenic spheroids, although not as extensive as that of microtic ones because of tissue availability, suggest a more even distribution of cartilage nodules, and formation of an organized multi-layered structure. This includes an inner region with low GAG content, a boundary with spindle like-cells, and an outer region with high GAG content and chondrogenic cells (Sox9 positive), which closely resemble the outer, inner perichondrium and chondrium of native cartilage, respectively (Figure 9B). In normal spheroids the inner region contained more cells than the outer region, and in microtic spheroids the inner region often formed off-centre beneath a thin outer region. Furthermore, such compartmentalization was never observed in chondrogenically differentiated ADSC spheroids of a comparable size. For these reasons, it seems unlikely that formation of the inner region is due to limited nutrient diffusion to the core of the spheroids rather than to a spontaneous ability of CSPCs to self-organize in 3D cultures. Finally, no cartilage hypertrophy-associated markers were detected either in normal or in microtic spheroids, ruling out the occurrence of chondrocyte hypertrophy and mineralization. Hence, it could be inferred that the spheroid system can be used to mimic the behavior of normal and abnormal ear cartilage.

## Conclusion

Together, our study suggests that in 3D spheroids, microtic and normal CSPCs undergo a chondrogenic differentiation process, which results in tissues morphologically similar to native microtic and normal cartilage, respectively. The similarity we have observed between microtic and normal CSPCs with their tissues of origin were not apparent in 2D cultures, which are therefore not sufficient to validate the suitability of microtic cells for cartilage engineering for auricle reconstruction. Furthermore, the similarity between microtic 3D spheroids and native microtic cartilage suggest they can provide a very valuable model to investigate cellular and molecular mechanisms underlying the disease. Recently, microtic ear-derived cells seeded in bioabsorbable scaffolds have been used for auricle reconstruction in patients (Zhou et al., 2018). However, more extended analysis is required to understand whether microtic cells will be able to maintain the appropriate biological and mechanical properties over time once the scaffold is degraded.

## Data Availability Statement

The raw data supporting the conclusions of this article will be made available by the authors, without undue reservation, to any qualified researcher.

## Ethics Statement

The studies involving human participants were reviewed and approved by NRES Committee London, UK – Fulham Research Ethics Committee (fetal tissue) and Camden and Islington Community Local Research Ethics Committee (pediatric tissue). Written informed consent to participate in this study was provided by the participants’ legal guardian/next of kin.

## Author Contributions

EZ designed and performed experiments, analyzed data, wrote the manuscript, and obtained funding. MB supervised research, obtained funding, and provided critical reading of the manuscript. NB obtained patient consent, collected tissues, and provided critical reading of the manuscript. PF supervised research, analyzed data, obtained funding, and wrote the manuscript. All authors contributed to the article and approved the submitted version.

## Conflict of Interest

The authors declare that the research was conducted in the absence of any commercial or financial relationships that could be construed as a potential conflict of interest.

## Footnotes

1 http://hdbr.org
