# Supplementary material for: Modeling Normal and Pathological Ear Cartilage in vitro Using Somatic Stem Cells in Three-Dimensional Culture
Source: Front Cell Dev Biol. 2020 Jul 28;8:666. doi: 10.3389/fcell.2020.00666 (PMC7402373; doi:10.3389/fcell.2020.00666)
Supplement: Supplementary file 1 [file Table_1.docx]

Supplementary Material

# Supplementary Table 1. List of tissues and cell lines used.

| **Cartilage origin type and name** | **Age** | **Gender** | **Cell line** | **Diagnosis** |
| --- | --- | --- | --- | --- |
| Microtic ear cartilage  31 | 9 years | F | hCSPC31 | Hemifacial microsomia |
| Microtic ear cartilage  32 | 12 years | M | hCSPC32 | Hemifacial microsomia |
| Microtic ear cartilage  34 | 9 years | F | hCSPC34 | Goldhenar syndrome |
| Microtic ear cartilage  36 | 10 years | unknown | hCSPC34 | Unilateral sporadic microtia, non syndromic |
| Microtic ear cartilage  42 | 16 years | M | NA | Unilateral sporadic microtia, non syndromic |
| Microtic ear cartilage  43 | 8 years | F | hCSPC42 | Goldhenar syndrome |
| Fetal ear cartilage  80 | 16 PCW | unknown | NA | NA |
| Fetal ear cartilage  81 | 22 PCW | unknown | NA | NA |
| Normal ear cartilage  90 | 9 years | M | hCSPC90 | Healthy donor (some Irregularity in the conceal bowl |
| Fat  11 | 16 years | M | hADSC | Hemifacial microsomia |
| Fat  12 | 16 years | M | hADSC | Gynecomastia |
| Fat  H20 | 12 years | F | hADSC | Parry Romberg syndrome / Facial atrophy |
| Fat  23 |  |  |  |  |
| Fat  33 | 14 years | F | hADSC | Lipoma |
| Fat  37 | 10 years | F | ADSC line | Hemifacial microsomia |
| Fat  40 | 17 years | M | ADSC line | Hemifacial microsomia |

F: female; M: male; C: cartilage; NA: not applicable; h: human; CSPC: cartilage stem precursor cells; ADSC: adipose-derived stem cells.

# Supplementary Table 2. Details of primers used for RT-PCR

| **Genes** | **Forward primer 5’ – 3’** | **Reverse primer 5’ – 3’** | **Annealing Temp (°C)** | **Product size (bp)** | **Cycles (RT-PCR)** |
| --- | --- | --- | --- | --- | --- |
| **hACAN**  **(Aggrecan)** | TACTCTGGGTTTTCGTGACTC | CGATGCCTTTCACCACGACTT | 56 | 450 | 32 |
| **hCol1**  **(Collagen 1)** | ATGCCTGGTGAACGTGGT | AGGAGAGCCATCAGCACCT | 56 | 87 | 35 |
| **hCol2**  **(Collagen 2)** | GCCTGGTGTCATGGGTTT | GTCCCTTCTCACCAGCTTTG | 56 | 71  125 | 35 |
| **hCol9**  **(Collagen 9)** | ACTGGGTTCTCTGGGTAGCC | ATGTGCTGATCTGTCGGTGC | 56 | 363 | 35 |
| **hCol10**  **(Collagen 10)** | AATCCCTGGACCGGCTGGAATTC | TTGATGCCTGGCTGTCCTGGAACC | 56 | 267 | 35 |
| **hELN**  **Elastin** | GAGTCGGAGTTGGAGGCATT | CAGGCACTGCTGCTCCATATTT | 58 | 639  582 | 35 |
| **hGAPDH** | TGATGACATCAAGAAGGTGGTGAAG | TCCTTGGAGGCCATGTGGGCCAT | 56 | 240 | 26 |
| **hNES**  **(Nestin)** | CAGCGTTGGAACAGAGGTTGG | TGGCACAGGTGTCTCAAGGGTAG | 56 | 389 | 35 |
| **hOSX**  **(Osterix)** | CGGGACTCAACAACTCT | CCATAGGGGTGTGTCAT | 56 | 308 | 35 |
| **hRPL19** | CAGGCTGTGATACATGTGGCG | GCGGAAGGGTACAGCCAAT | 56 | 130 | 26 |
| **hRunx2**  **(Runt-related transcription factor 2)** | GCACAGACAGAAGCTTGAT | CCCAGTTCTGAAGCACCT | 56 | 417  351 | 35 |
| **hSox9**  **(SRY-Box 9)** | CTACGACTGGACGCTGGTGC | CGATGTCCACGTCGCGGAAG | 56 | 578 | 35 |
| **hVIM**  **(Vimentin)** | GACACTATTGGCCGCCTGCAGGATGAG | CTGCAGAAAGGCACTTGAAAGC | 56 | 418 | 35 |

# Supplementary Table 3. Antibodies and dyes used for protein detection and nuclear staining.

| **Antibody** | **Host / Class** | **Dilution** | **Company and cat. number** |
| --- | --- | --- | --- |
| **Primary** | | | |
| **Aggrecan** | Rabbit, polyclonal | 1:100 | Santa Cruz, sc-25674 |
| **Collagen 1** | Rabbit, polyclonal | 1:100 | Novus Biologicals,  NB600-408 |
| **Collagen 2** | Rabbit, polyclonal | 1:150 (IF/IHC)  1:500 (WB) | Abcam, ab34712 |
| **Elastin** | Mouse, monoclonal | 1:100 | AbCam, AB21599 |
| **Nestin** | Rabbit, polyclonal | 1:500 | Millipore, ABD69 |
| **Sox 9** |  |  |  |
| **Vimentin** | Mouse, monoclonal | 1:500 | Dako, M0725 |
| **Alkaline Phosphatase** | Mouse, monoclonal | 1:200 | Hybridoma bank DSHB |

| **Secondary** | | | |
| --- | --- | --- | --- |
| **Anti-rabbit Alexa Fluor^®^ 594** | Donkey | 1:400 | Molecular probe, Invitrogen A21203 |
| **Anti-mouse Alexa Fluor^®^ 488** | Donkey | 1:400 | Molecular probe, Invitrogen A21202 |
| **Anti-mouse / Biotinylated** | Goat | 1:400 | Dako, E0433 |
| **Anti-rabbit / Biotinylated** | Goat | 1:400 | Dako, E0432 |
| **Anti-rabbit / HRP conjugated** | Goat | 1:4000 | Dako, P0448 |
| **Dye** | | | |
| **Hoechst 33258** |  | 1:800 | Invitrogen, H1398 |

# Supplementary Table 4. Diameter of normal spheroids cultured in either standard proliferation (undifferentiated) or chondrogenic (differentiated) media.

|  | **4 weeks** | | **6 weeks** | |
| --- | --- | --- | --- | --- |
|  | **Undifferentiated (mm)** | **Differentiated**  **(mm)** | **Undifferentiated**  **(mm)** | **Differentiated**  **(mm)** |
|  | 1 | 2.47 | 0.581 | 1.97 |
|  | 1.01 | 2.39 | 0.647 | 2.277 |
|  | 1 | 2.48 | 0.622 | 1.956 |
|  | **-** | **-** | 0.637 | 2.011 |
| **Mean (mm)** | 1.003 | 2.447 | 0.622 | 2.054 |
| **St. Dev** | 0.006 | 0.049 | 0.029 | 0.151 |
| **± SEM** | 0.003 | 0.028 | 0.015 | 0.075 |

# Supplementary Table 5. Number of cartilage nodules identified in close proximity to the boundary or interrupting the boundary.

|  | **Normal** | | | **Microtic** | | | | | | |
| --- | --- | --- | --- | --- | --- | --- | --- | --- | --- | --- |
| **Cell line** | **90** | | | **31** | | | **32** | | | **34** |
| Spheroid 1 | 0 | 0 | 0 | 3 | 2 | 2 | NA | | | NA |
| Spheroid 2 | 0 | 0 | 0 | 0 | 2 | 1 | NA | | | NA |
| Spheroid 3 | 0 | 0 | 0 | 0 | 0 | 0 | 9 | 8 | 3 | NA |

For each Spheroid, 3 individual sections were analysed. NA: not applicable, boundary not identifiable.
