# Supplementary figures and images for: Modeling Normal and Pathological Ear Cartilage in vitro Using Somatic Stem Cells in Three-Dimensional Culture
Source: Front Cell Dev Biol. 2020 Jul 28;8:666. doi: 10.3389/fcell.2020.00666 (PMC7402373; doi:10.3389/fcell.2020.00666)

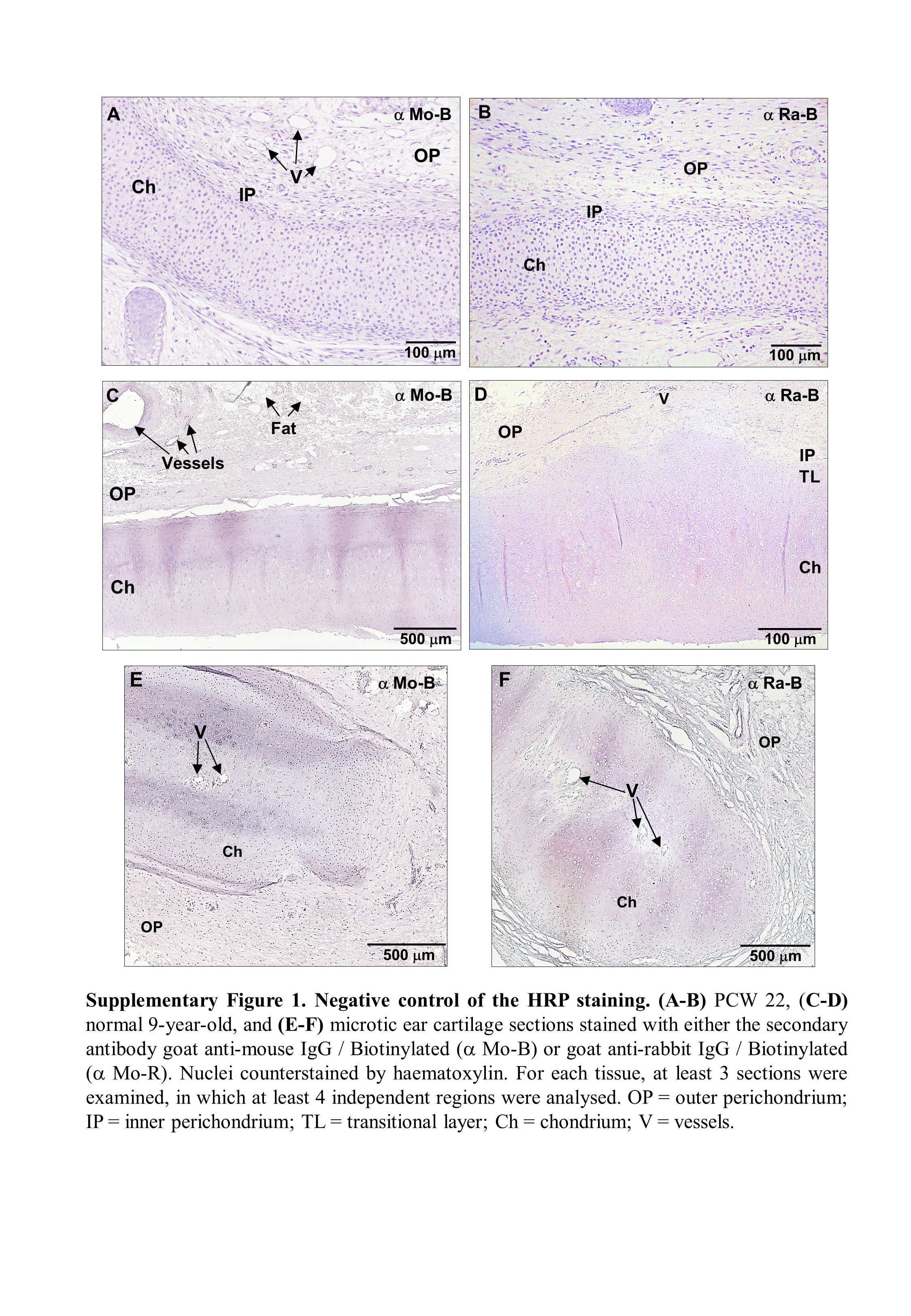

Supplement: Supplementary file 2 [file Image_1.JPEG]

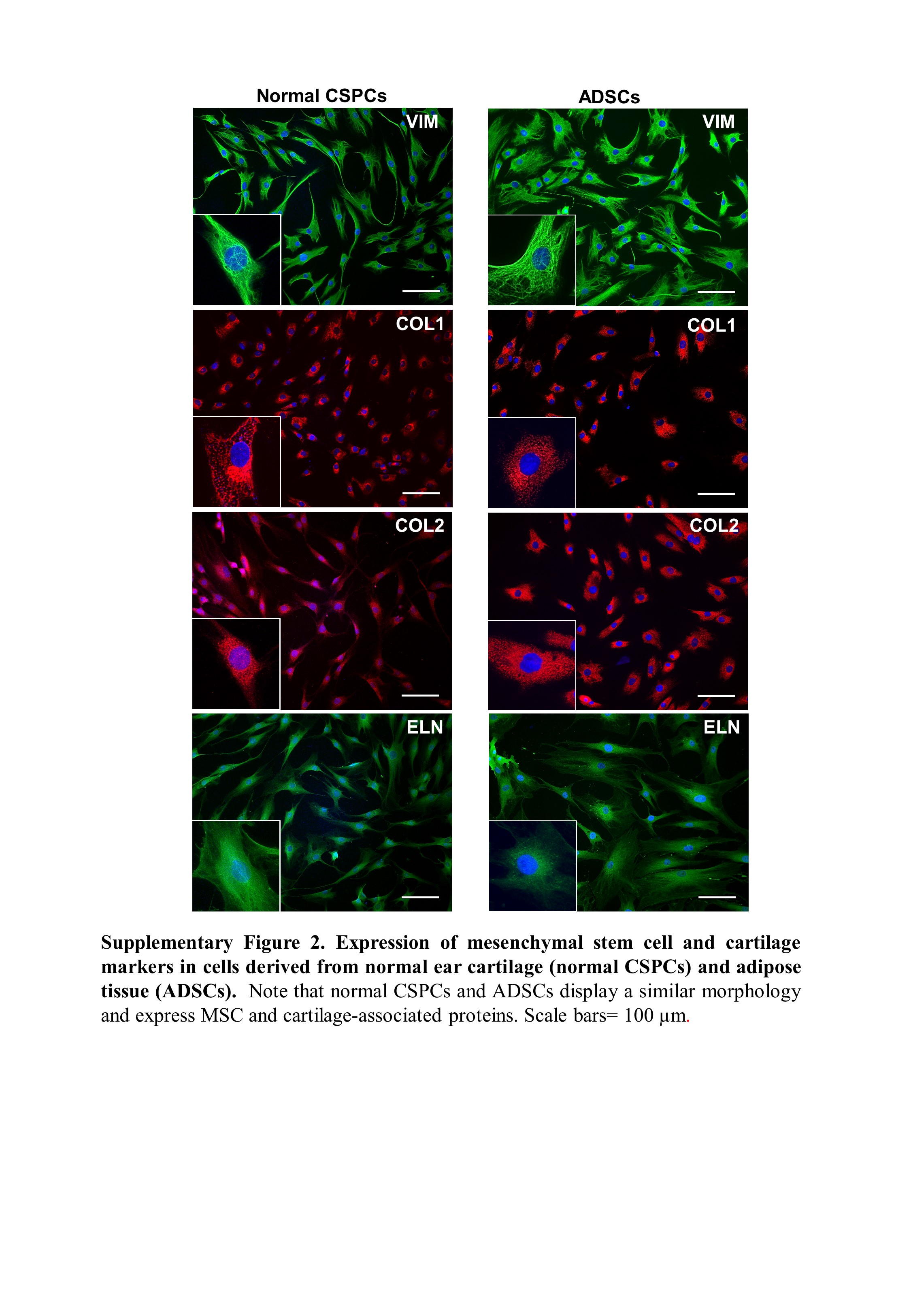

Supplement: Supplementary file 3 [file Image_2.JPEG]

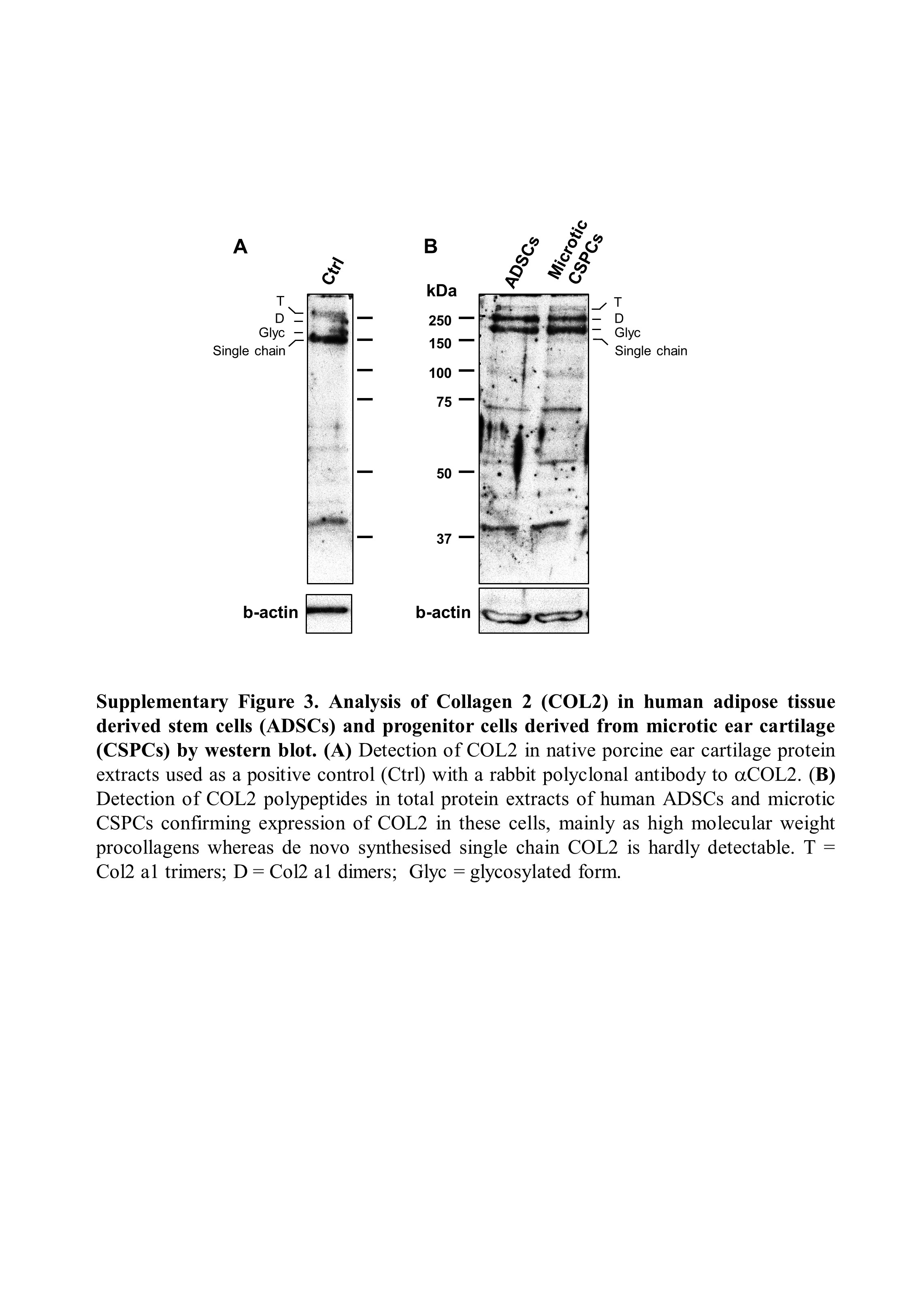

Supplement: Supplementary file 4 [file Image_3.jpg]

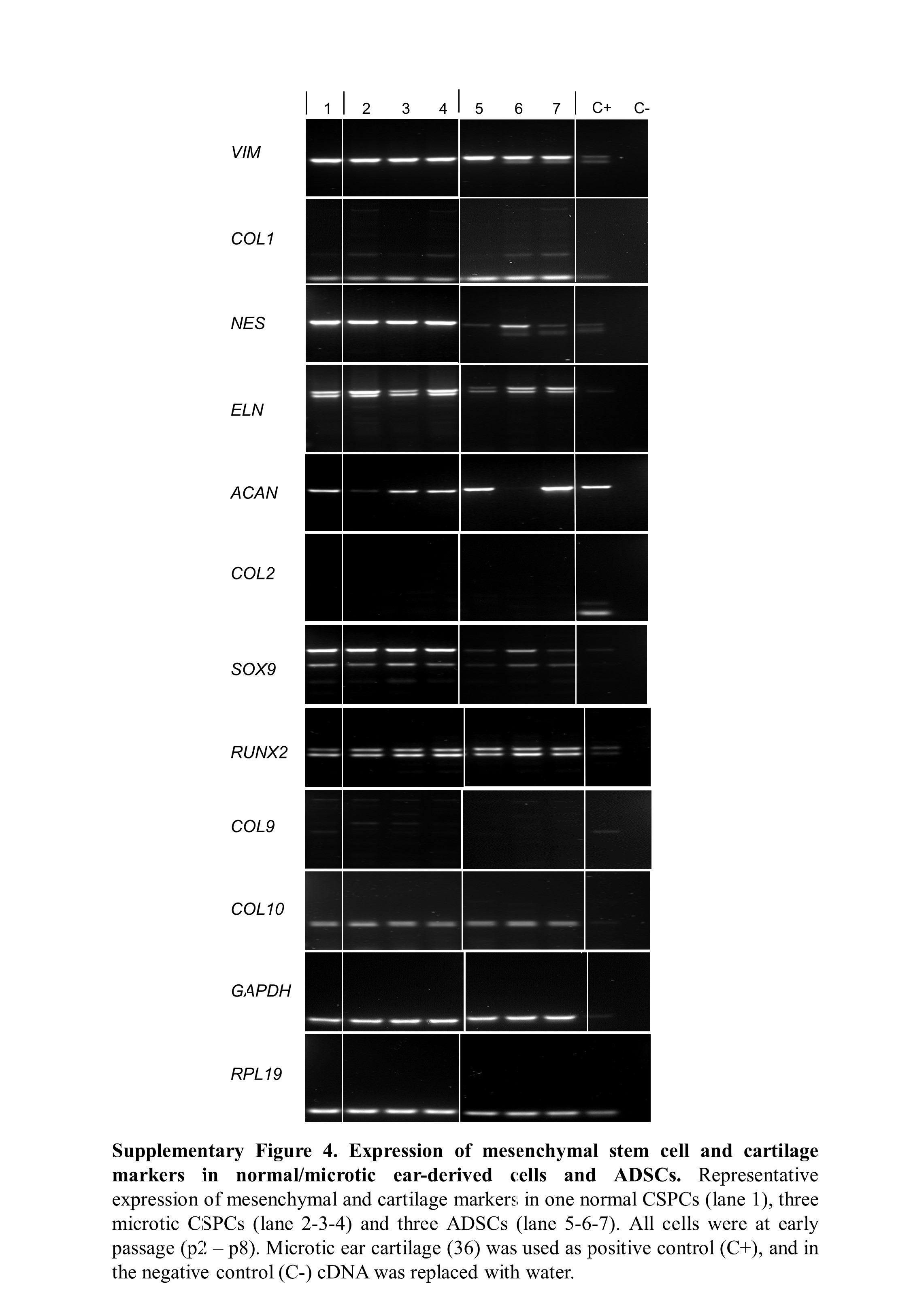

Supplement: Supplementary file 5 [file Image_4.JPEG]

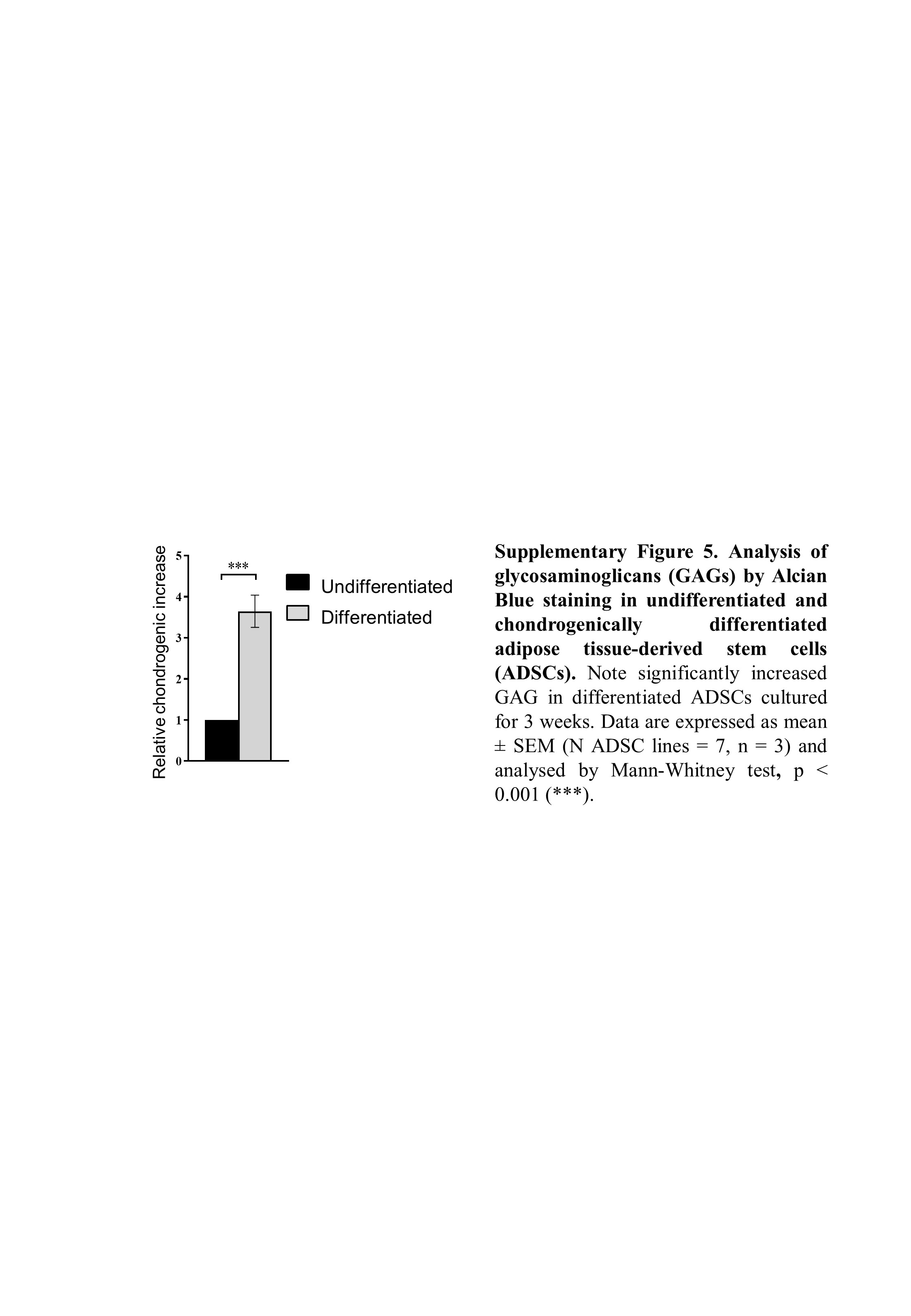

Supplement: Supplementary file 6 [file Image_5.JPEG]

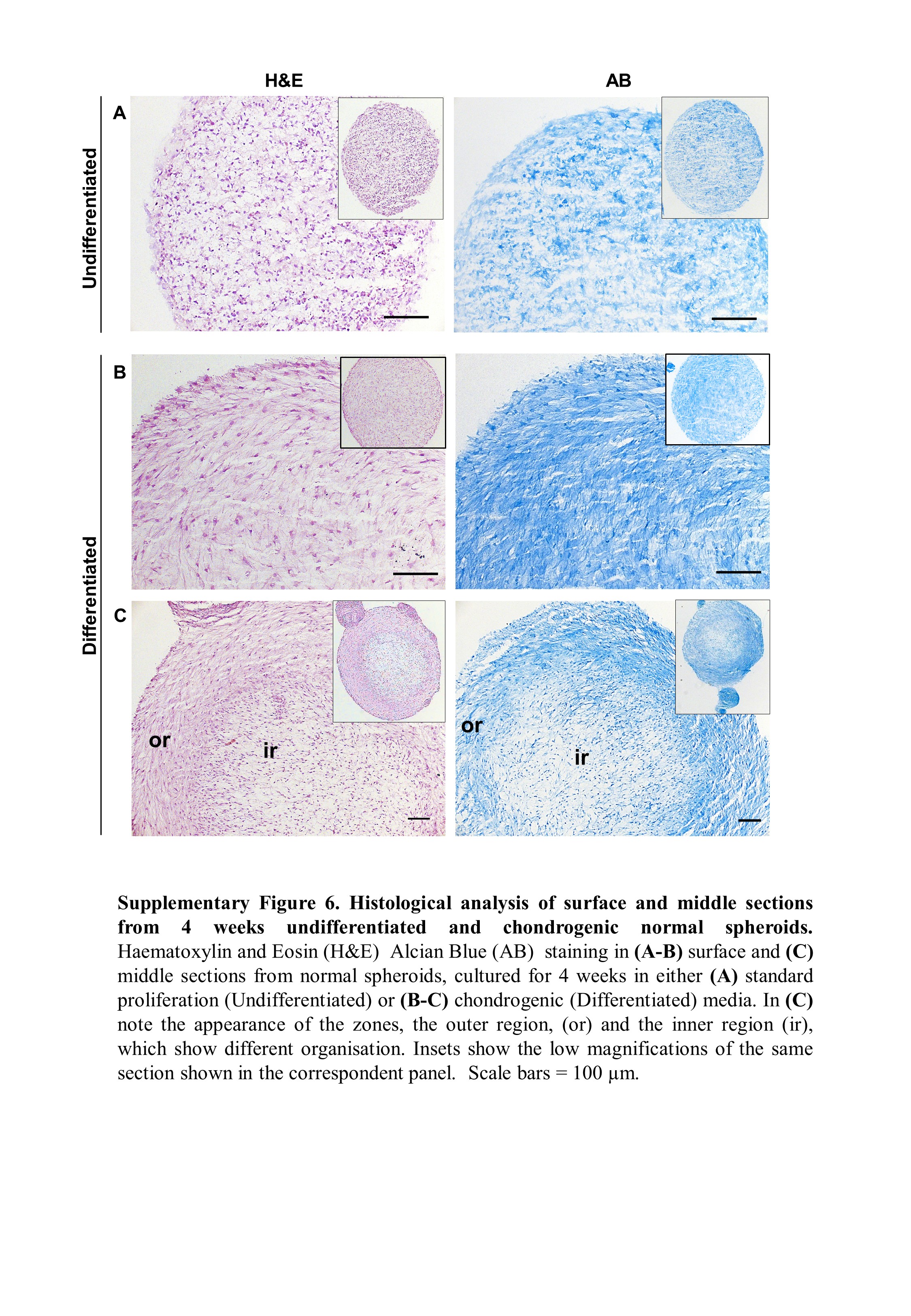

Supplement: Supplementary file 7 [file Image_6.JPEG]

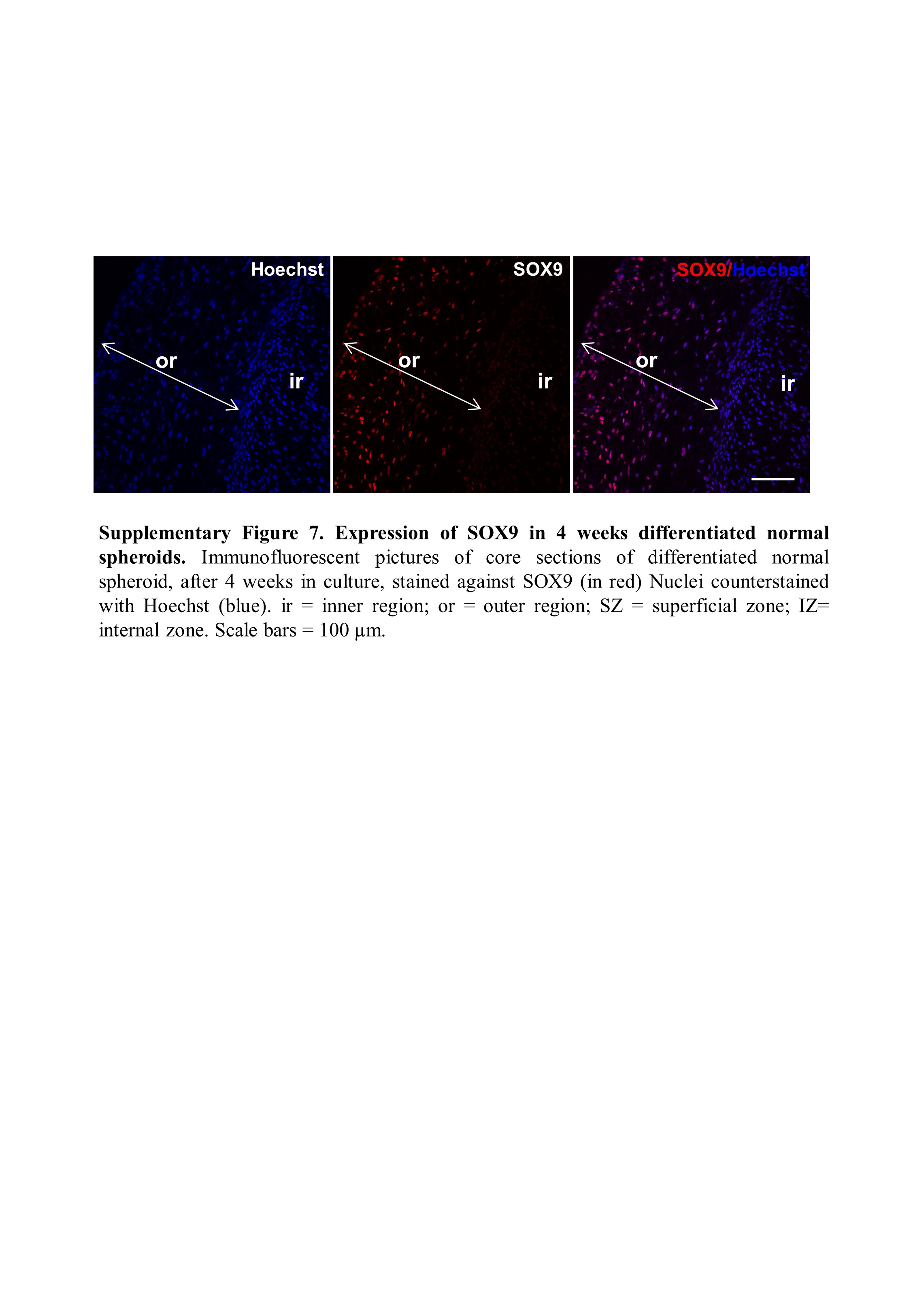

Supplement: Supplementary file 8 [file Image_7.JPEG]

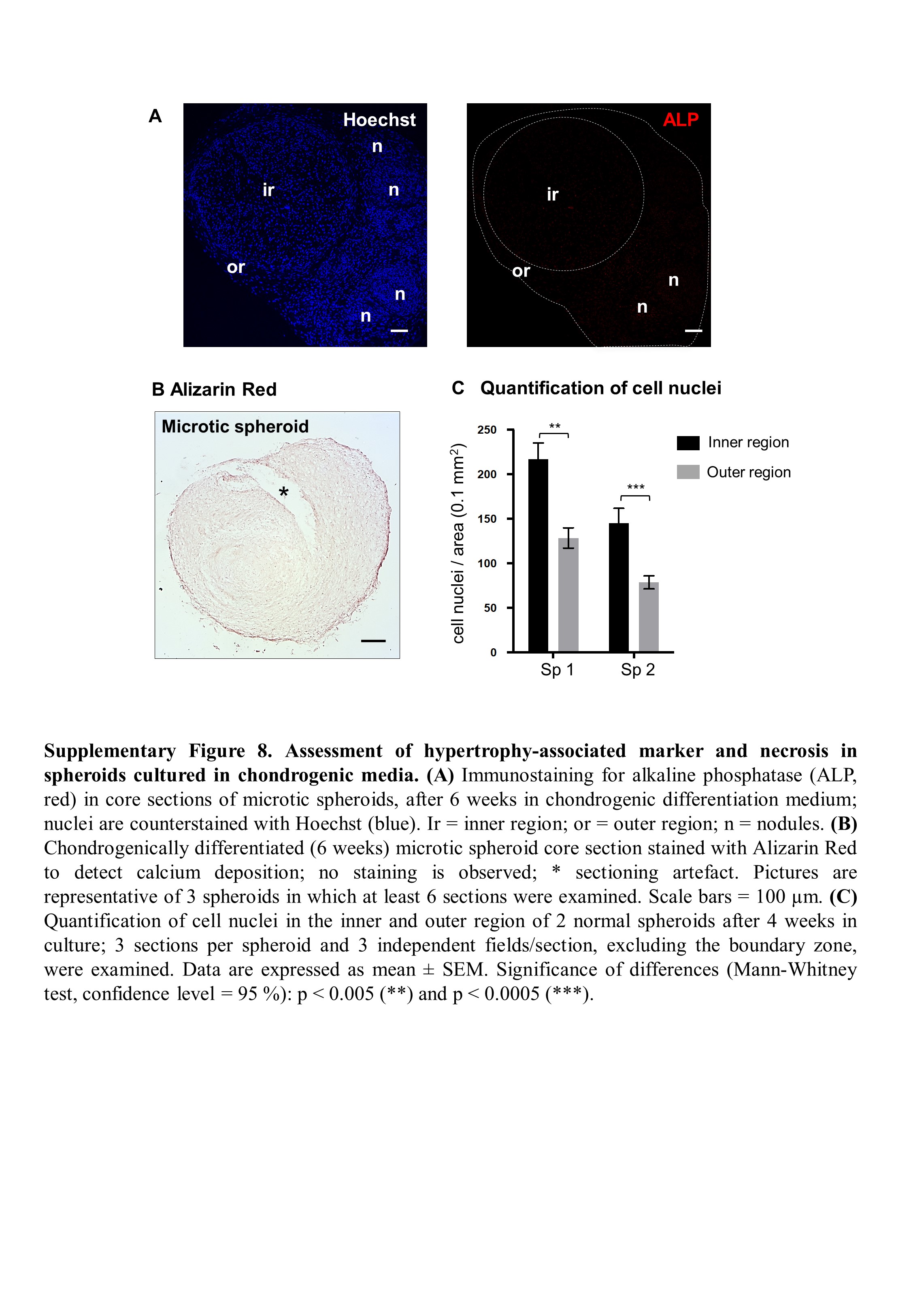

Supplement: Supplementary file 9 [file Image_8.jpg]

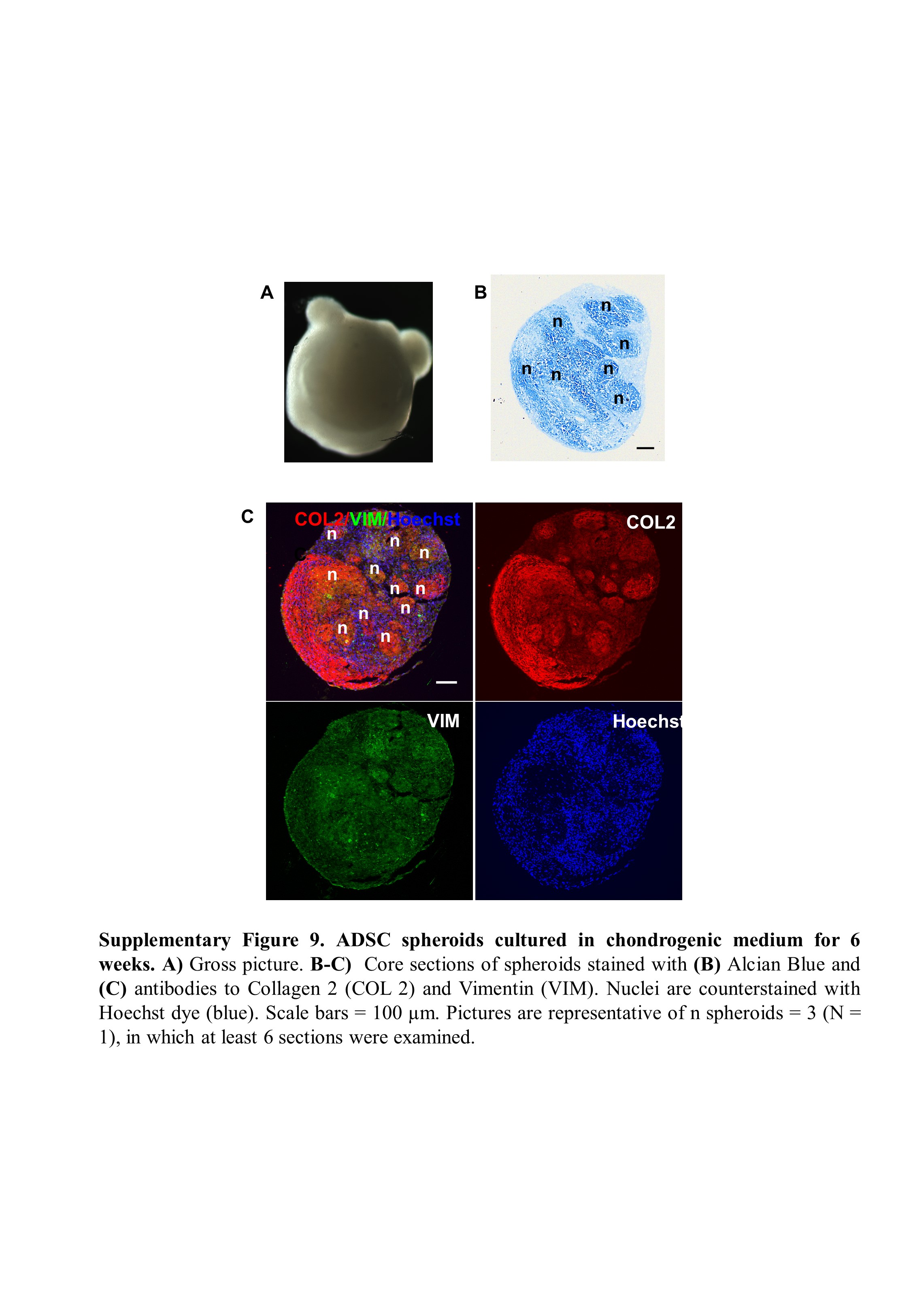

Supplement: Supplementary file 10 [file Image_9.JPEG]
