# Supplementary material for: Modeling Normal and Pathological Ear Cartilage in vitro Using Somatic Stem Cells in Three-Dimensional Culture
Source: Front Cell Dev Biol. 2020 Jul 28;8:666. doi: 10.3389/fcell.2020.00666 (PMC7402373; doi:10.3389/fcell.2020.00666)
Supplement: Supplementary file 11 [file Data_Sheet_1.pdf]

Full Scan of agarose gels of PCR products shown in Supplementary Materials

1 Reverse Transcription-PCR gel blots

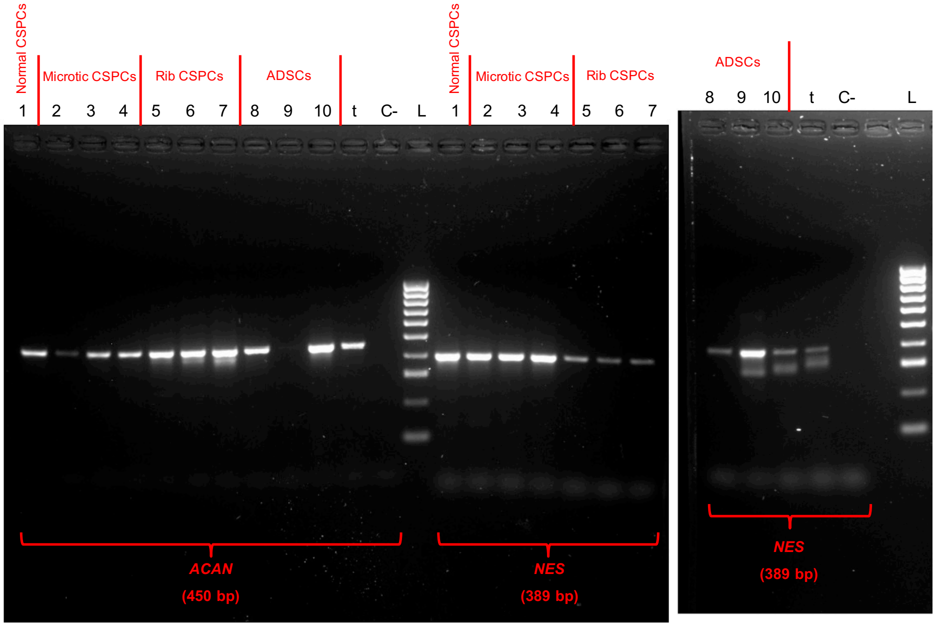

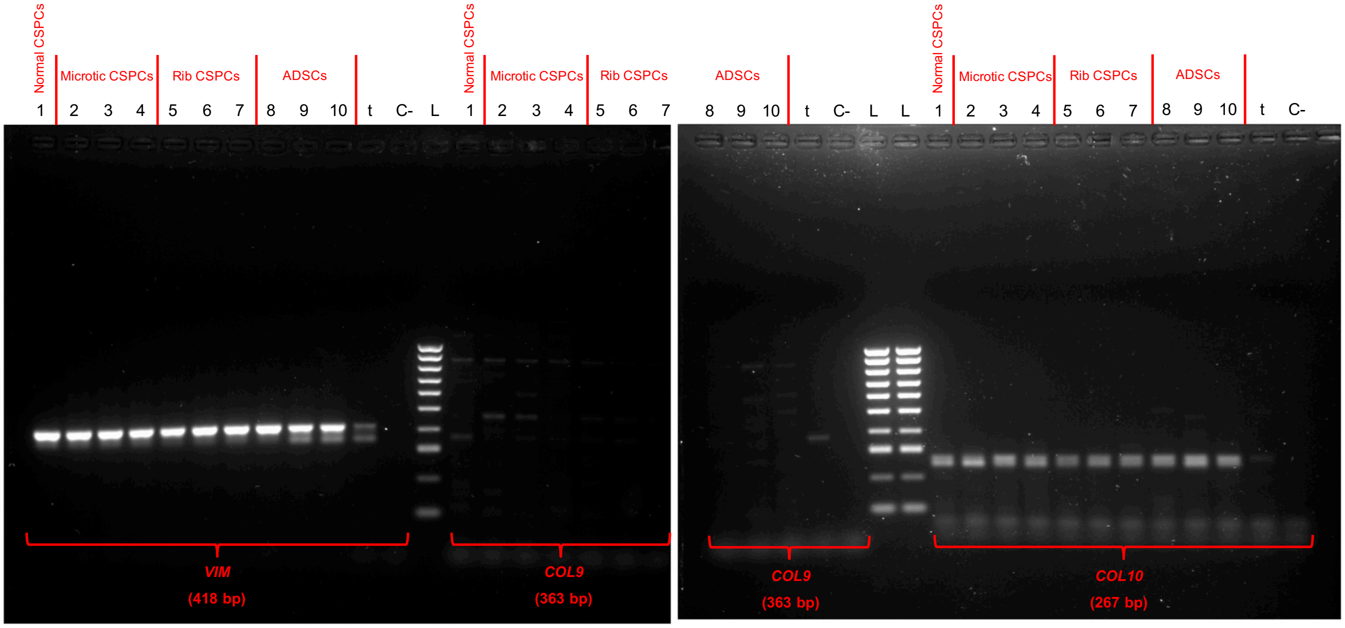

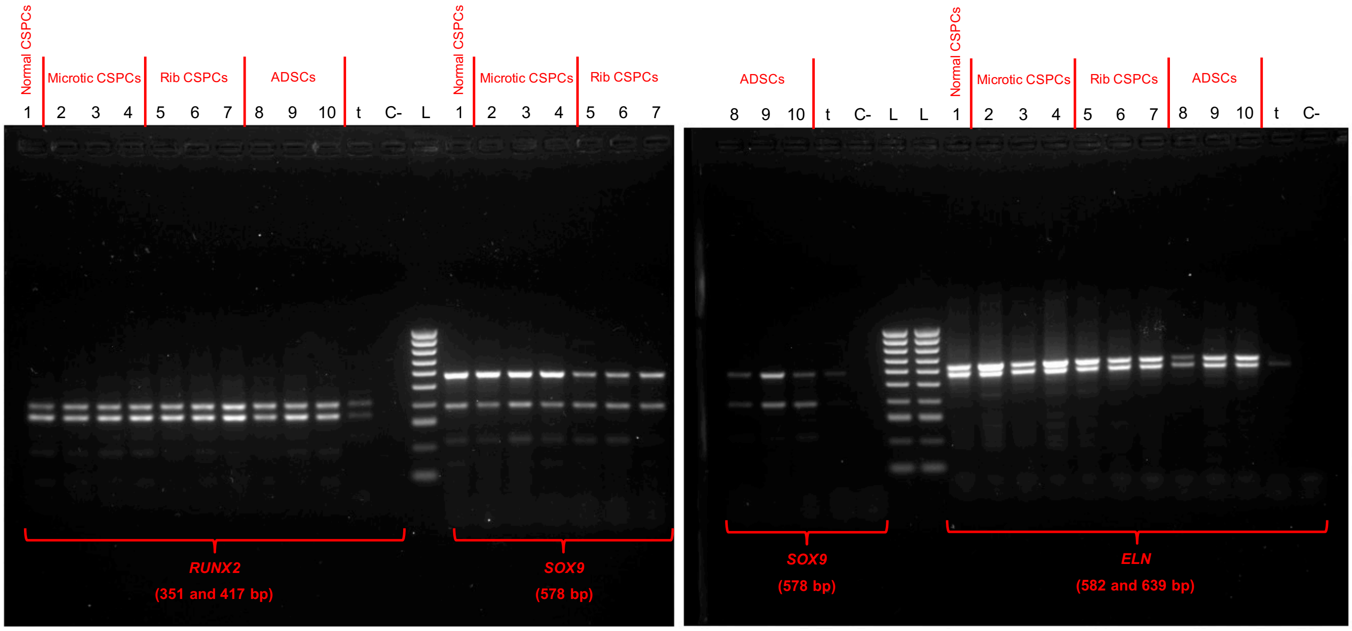
Normal CSPCs
Microtic CSPCs
Rib CSPCs
ADSCs
1 2 3 4 5 6 7 8 9 10 t C- L 1 2 3 4 5 6 7
RUNX2
(351 and 417 bp)
SOX9
(578 bp)
ADSCs
Normal CSPCs
Microtic CSPCs
Rib CSPCs
ADSCs
8 9 10 t C- L L 1 2 3 4 5 6 7 8 9 10 t C-
SOX9
(578 bp)
ELN
(582 and 639 bp)

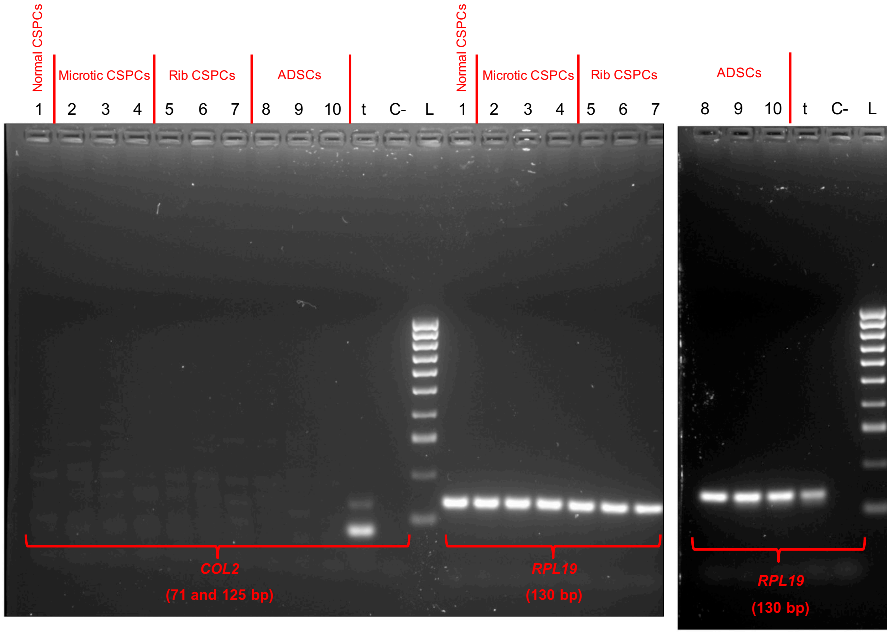
Normal CSPCs
Microtic CSPCs
Rib CSPCs
ADSCs
1 2 3 4 5 6 7 8 9 10 t C- L 1 2 3 4 5 6 7
COL2
(71 and 125 bp)
RPL19
(130 bp)
ADSCs
8 9 10 t C- L
RPL19
(130 bp)

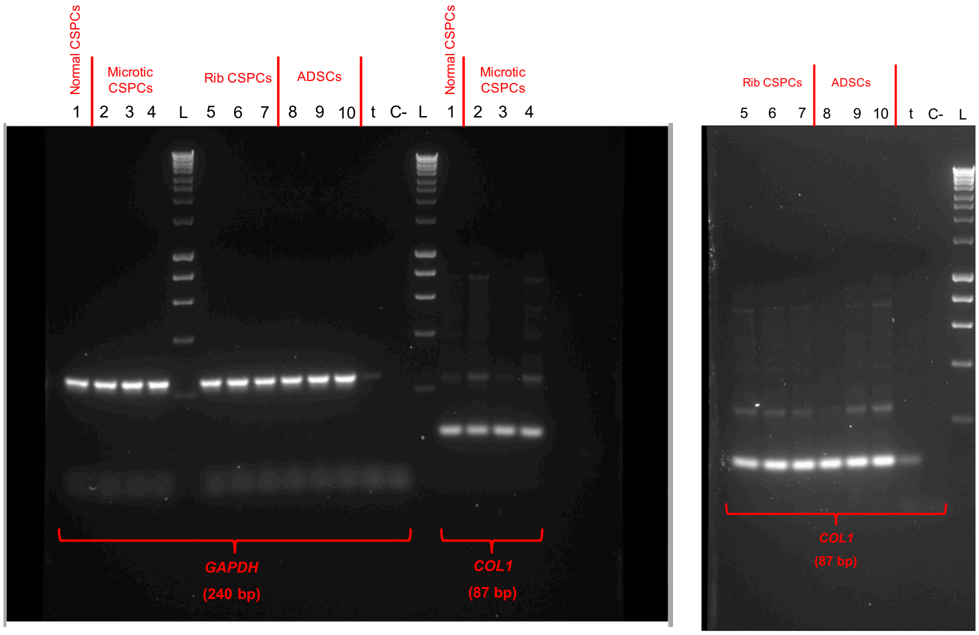

Supplementary Material 2. **Full scan of entire original PCR gels shown in Supplementary Fig. 4.** (1) one normal CSPCs, (2-4) three microtic CSPCs, (5-7) three rib-CSPCs, and (8-10) three ADSCs. Aggrecan (*ACAN*), Nestin (*NES*), Vimentin (*VIM*), Collagen 9 (*COL9*), Collagen 10 (*COL10*), Runt-related transcription factor 2 (*RUNX*), SRY-Box (*SOX9*), Elastin (*ELN*), Collagen 2 (*COL2*),Ribosolmal Protein L19 (*RPL19*), GAPDH (*GAPDH*) and Collagen 1 (*COL1*)genes. Microtic ear cartilage (t) was used as positive control and in the negative control (C-) cDNA was replaced with water. Hyperladder 100 bp was used in all gels, except in *GAPDH* and *COL1* gels (Hyperladder 1 Kb).
